# Overall Downregulation of mRNAs and Enrichment of H3K4me3 Change Near Genome-Wide Association Study Signals in Systemic Lupus Erythematosus: Cell-Specific Effects

**DOI:** 10.3389/fimmu.2018.00497

**Published:** 2018-03-13

**Authors:** Zhe Zhang, Lihua Shi, Li Song, Kelly Maurer, Michele A. Petri, Kathleen E. Sullivan

**Affiliations:** ^1^The Center for Biomedical Informatics, The Children’s Hospital of Philadelphia, Philadelphia, PA, United States; ^2^The Division of Allergy Immunology, The Children’s Hospital of Philadelphia, Philadelphia, PA, United States; ^3^Division of Rheumatology, Johns Hopkins University School of Medicine, Baltimore, MD, United States

**Keywords:** epigenetic, H3K4me3, ATF3, lupus, gene expression, RNA-seq, chromatin immunoprecipitation-seq, genome-wide association study

## Abstract

This study was designed to define gene expression and H3K4me3 histone modifications in T cells, B cells, and monocytes in systemic lupus erythematosus (SLE). Array studies of total peripheral blood mononuclear cells have demonstrated gene expression signatures related to neutrophils, interferon, and other inflammatory pathways. It is not clear how consistent these effects are across different cell types. In this study, RNA-seq and chromatin immunoprecipitation-seq were utilized to identify gene expression patterns and H3K4me3 histone modifications related to promoter activation in SLE. Across the three cell types, there was 55% concordance for gene expression changes related to SLE. Key conserved pathways were ribosome biogenesis among upregulated genes and heat shock response among downregulated genes. ETS family transcription factors (TFs) and STAT1 were revealed as common regulators by position weight matrices. When epigenetic changes were leveraged with gene expression, the pivotal TFs ATF3 and FOS were defined with ATF3 also cross-referencing with gene expression-identified TFs. Genome-wide association study (GWAS) single nucleotide polymorphisms associated with SLE were cross-referenced with both mRNA and H3K4me3 changes in SLE. Baseline mRNA expression and H3K4me3 peak height was higher at sites that cross-referenced with GWAS signals, however, all three cell types exhibited an overall decrease in expression of GWAS-associated RNAs differentially expressed in SLE. H3K4me3 changes in SLE were also enriched in GWAS-associated sites. In summary, the SLE disease process is associated with both shared and cell-specific changes in gene expression and epigenetics. Surprisingly, GWAS-associated RNAs were overall markedly decreased across all three cell types. TF analysis identified ATF3, FOS, STAT1, and ETS family members as critical, all pathways with a recognized relationship to the SLE disease process. GWAS signals clearly mark both cell-type specific changes in SLE as well as concordant changes across all three cell types. Interpretation of single nucleotide polymorphism effects in SLE will require tissue-specific mechanistic studies and therapeutics will require mechanistic studies in multiple cell types.

## Introduction

Systemic lupus erythematosus (SLE) is the quintessential systemic autoimmune disease. Nearly, every organ can be involved in SLE and a great diversity of immunologic features have been described (1). In spite of enormous effort to identify a common pathway that links all clinical and laboratory features, only the interferon signature has been identified consistently across multiple laboratories (2–4). This major advance in the understanding of SLE arose from the study of peripheral blood mononuclear cells (PBMC) by gene expression arrays and is associated with a broad range of autoimmune diseases (5–7). Most cells express type I interferon receptors, although transcriptional responses differ between cell types. Thus, responses to interferons could be coordinate among different cells or discordant between cell types. Other cytokines over-expressed in SLE have receptors that are not ubiquitously expressed. Furthermore, we have identified high levels of circulating endotoxin in SLE which could drive immune activation in a cell-specific manner due to the limited expression of TLR4 (8). We therefore hypothesized that different cell types in peripheral blood are impacted by the SLE disease process in both coordinate and distinct manners and key commonalities could be dissected by using a joint transcriptomic and epigenetic approach in purified cell types. Recognition of distinct cell effects is critical for interpretation of genome-wide association studies (GWASs) where efforts to examine mechanism may require a tissue-specific approach.

Several prior studies have used array approaches on purified hematopoietic cells. In these studies, concordance across cell types was modest (9–11). Interferon response genes exhibited increased expression as a category in all sorted cell types but gene-level expression was often tissue specific. Using RNA-seq, deconvolution was applied, finding that MHC gene expression was most altered in B cells and ribosomal gene expression was most altered in monocytes (12). Thus, there has been little concordance across cell types in different studies. These expression studies suggest that many SLE effects are tissue specific; however, these studies have generally used arrays with analyses restricted to the genes included on the array. RNA-seq offers a more holistic approach and has the advantage of a greater dynamic range.

A compelling reason to focus on epigenetic changes is that these changes are typically more stable in patients over time compared with gene expression. Although epigenetic changes in SLE have received attention in recent years, most studies have focused on DNA methylation (13–16). Changes in histone modifications are restricted to tighter peaks than that seen with DNA methylation and can be more easily used to focus on DNA motifs associated with transcription factor (TF) binding. We have previously found IRF1 as a pivotal TF by defining H4ac peaks in monocytes from SLE patients (17–19). We went on to define changes in H3K4me3 at promoters and enhancers in SLE monocytes, linking a type I interferon effect to chromatin and defining a set of TFs identified through position weight matrices (PWMs) (20, 21). There are additional studies that have utilized ATAC-seq of SLE B cells, which found that increased accessibility was seen at loci related to B cell activation (22). To best define cell-type specific changes in the transcriptome and epigenome in SLE, we chose to examine changes in H3K4me3, a histone modification associated with active promoters. We paired this with RNA-seq to define changes in gene expression. Finally, we cross-referenced our SLE effects on the transcriptome and epigenome with published GWAS variants. Our findings support a model where many SLE effects are concordant with cell-type specific differences in H3K4me3 and enrichment of GWAS signals. These data have important implications for mechanistic studies of single nucleotide polymorphisms and the development of therapeutics.

## Materials and Methods

### Samples and Cells

The female healthy donor blood samples were obtained from the Center for AIDS Research, under a protocol approved by the University of Pennsylvania Perelman School of Medicine Institutional Review Board (IRB) in accordance with their guidelines. All subjects gave written informed consent in accordance with the Declaration of Helsinki. Samples were handled identically as the shipped SLE samples. The female SLE patient blood samples were from the Johns Hopkins Lupus Cohort (23, 24) under a separate IRB protocol in accordance with the recommendations and guidelines of the Johns Hopkins IRB Committee. All patients gave written informed consent in accordance with the Declaration of Helsinki. Six SLE patients and six controls were used (Table 1). PBMC and monocytes were purified using adherence as previously described (8). Dynal beads (Dynabeads, Invitrogen, Oslo, Norway) were used to separate CD3 T cells and CD19 B cells according to the manufacturer’s protocol. Flow cytometry was used to ensure purity of the three cell populations.

**Table 1:** Clinical characteristics of patients.

Patient	Assays	Autoantibodies	SLEDAI	PGA	Medications
1,077	RNA-seq, ChIP-seq	dsDNA, Sm, RNP	0	1.4	MMF, ASA
985	RNA-seq, ChIP-seq	dsDNA	2	0.5	HCQ, NSAID
1,169	RNA-seq, ChIP-seq	dsDNA	0	0.5	HCQ, NSAID, ASA
2,280	ChIP-seq	dsDNA, Sm, Ro	6	0.5	2.5 mg prednisone, MMF, HCQ
310	ChIP-seq	dsDNA	3	0.5	HCQ
2,239	ChIP-seq	dsDNA, Sm, Ro, RNP	16	2.5	HCQ

*HCQ, hydroxychloroquine; NSAID, non-steroidal anti-inflammatory; MMF, mycophenolate mofetil*.

Peripheral blood mononuclear cells were stimulated with 3 µg/ml of phytohemagglutinin (PHA) for 24 h followed by cell separation as above. The MYC inhibitor, 10058-F4, was used at a concentration of 50 µM. The inhibitor led to minimal cell death at this concentration and was added 20 min prior to stimulation with PHA.

### RNA Isolation and RNA-Seq

Total RNA was isolated from isolated T cells, B cells, and monocytes by the Qiagen RNeasy Kit (Valencia, CA, USA) and DNA was removed by column DNase digestion. The RNA quality was defined by RIN and OD260/280. The RNA-seq libraries were made with the Ovation^®^ Ultralow Library Systems and sequenced on an Illumina HiSeq at BGI@CHOP. A customized workflow was utilized. We aligned sequence reads to the human reference genome (GRCh38) and transcriptome using the STAR program. Read counts of each cell type were converted to log2 scale and normalized by the Lowess method. The limma method was applied to the normalized data to obtained differentially expressed genes.

### Chromatin Immunoprecipitation (ChIP) and ChIP-Seq

Chromatin immunoprecipitation assay experiments were performed as previously described (20, 25). The antibody for H3K4me3 was from Active Motif (Carlsbad, CA, USA). The Illumina TruSeq ChIP library preparation kit (San Diego, CA, USA) was used and sequencing was performed on an Illumina HiSeq. The reference genome (hg38) was indexed by the *novoindex* function of the *NovoAlign* package. Reads were filtered by *SAM* fields such as *mapq, cigar*, and *flag* and extended to 200 bp long at their 3′ end. Reads were mapped to the 1 kb promoter regions of known genes to get an integer matrix from each cell type. The read count matrices were normalized and analyzed the same way as the RNA-seq data to obtain results of differential H3K4me3.

### Gene Set Analysis

Differential expression or differential H3K4me3 of genes was ranked by their significance using the limma test. Parametric analysis of gene set enrichment (PAGE) method was used to compare each gene set and all the other genes. PAGE reported the significance of the difference with a *p*-value, false discovery rate, and enrichment score. Positive and negative enrichment scores indicated the overall increase and decrease, respectively, of gene expression or H3K4me3 of the gene set.

### TF Analysis

2,414 PWMs of TFs from multiple databases (ENCODE, Jasper, TRANSFAC, and UniPROBE) were used as the reference dataset. To identify TF targets, we mapped promoter sequences of known genes to these PWMs and selected genes with the best matches to a PWM as potential target genes regulated by the corresponding TF. On average, each target set includes approximately 1,300 genes. We ran gene set analysis to identify target sets with overall change of expression or H3K4me3 in SLE.

### Genome-Wide Association Cross-Referencing

Chromatin immunoprecipitation-seq data processed as above were used to analyze enrichment around SNP sites. The average coverage around a 1 kb (−500 to +500) region of SNPs was calculated. The log-transformed coverage was normalized by Loess method between all samples within each cell type. RNA-seq data were processed as previously described. SNPs were mapped to nearest gene TSS for RNA-seq analysis. Gene-level read counts were normalized and group comparison between controls and patients were also done as previously described. 95 SNP with GWAS *p*-values less than 1E-8 were selected from a study of 1,311 SLE patients (26). Additionally, a set of 46 SNPs (27) curated from two published studies was used (28, 29) and this is referred to as LD46 and a set of 25 SNPs curated from a meta-analysis of 7,219 SLE cases was used and referred to as META25 (28). All three sets of SNPs were used to generate a joint set of 143 SNPs used for analyzing RNA-seq data. The ChIP-seq data were analyzed using all SNPs and binning according to the *p*-value of the association with SLE.

Online visualization of data is provided at http://projectsle2.awsomics.org.

## Results

### Transcriptomic Analysis

**Figure 1:**
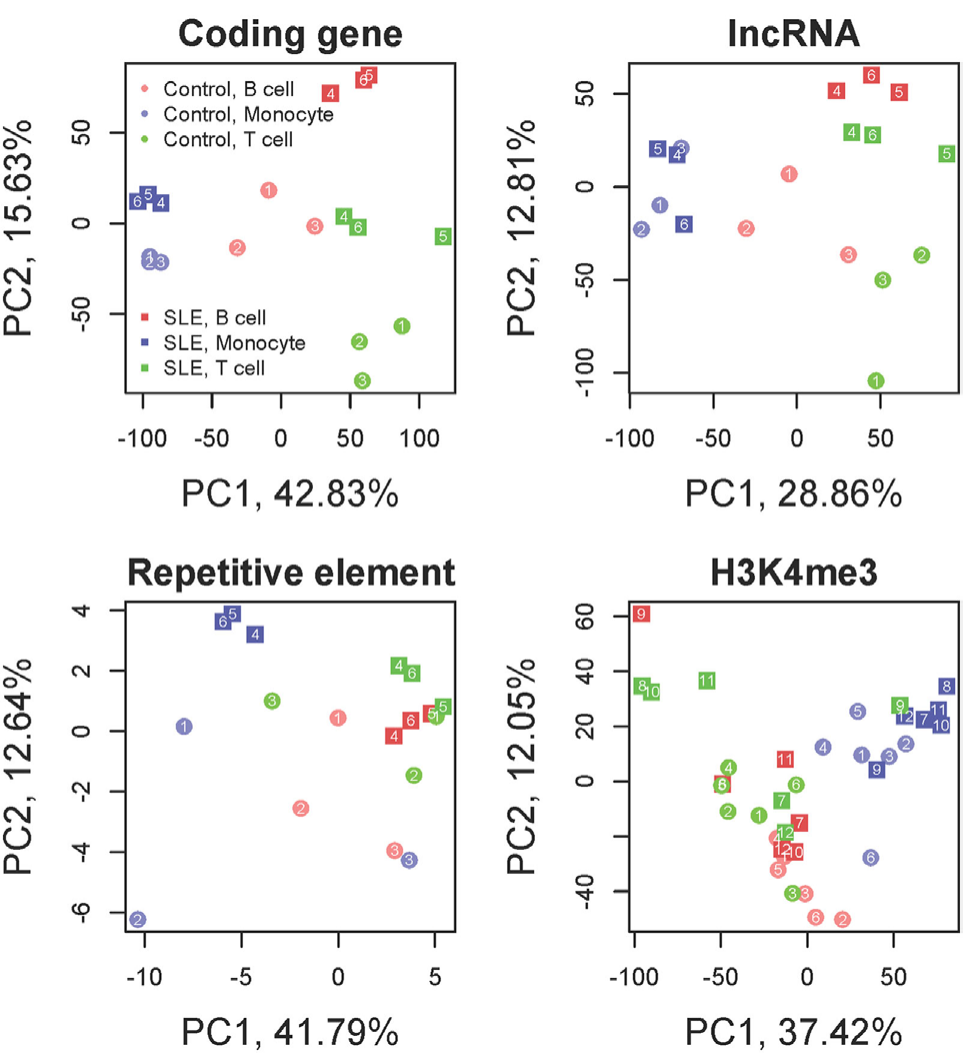
Principal component analysis. Coding genes, long non-coding RNAs (lncRNA), repetitive elements and H3K4me3 are displayed. The colors distinguish the three cell types. The shapes distinguish systemic lupus erythematosus (SLE) from control. Samples segregate by cell type and by disease state in all analyses. Each SLE-control cell type pair exhibits a uniform shift in the PC2 axis from control to SLE while the PC1 axis largely discriminates cell type for coding genes and lncRNAs. Repetitive elements still exhibit clear distinctions between cell type and disease state but the direction of change is different from the coding genes. H3K4me3 has a strong cell type dependence but less discrimination between disease and control samples.

There is relatively little known about the effect of SLE in different cell types and whether the interferon effect is common to all hematopoietic cell types. The goal of this transcriptomic analysis was to identify genes with differential expression between control and SLE groups that were concordant in CD3 T cells, CD19 B cells, and monocytes. Control and SLE samples clearly clustered distinctly in a principal component analysis, demonstrating a clear effect of disease (Figure 1). The direction of change for SLE compared with control was the same for the three cell types. Coding genes and long non-coding RNAs exhibited similar patterns while repetitive elements were different.

#### Differential Expression in Each Cell Type

In CD19 B cells, 1,589 and 1,489 genes had higher and lower expression, respectively in SLE (at least twofold change and *p* < 0.05). “Ribosome biogenesis” (GO:0042254) was the most upregulated gene set and the MSigDB “TNFa signaling *via* NFKB” pathway was the most downregulated gene set. In CD3 T cells, 1,313 and 1,173 genes had, respectively, higher and lower expression in SLE. “Ribosome biogenesis” and “TNFa signaling *via* NFKB” pathway were also the most up- and downregulated gene sets, respectively, as was seen in B cells. In monocytes, 848 and 716 genes had higher and lower expression in SLE, respectively. “Ribosome biogenesis” was again the most upregulated gene set and “nuclear chromatin” (GO:0000790) was the most downregulated gene set.

From this initial comparison, two findings stand out. (1) Upregulated and downregulated gene sets were quantitatively balanced in all three cell types. (2) Surprisingly, the gene set “ribosome biogenesis” was identified as upregulated in all three cell types while heat shock protein genes were uniformly found to be downregulated in each cell type. Individual genes differed; however, the signatures were robust across cell types.

#### Concordance of Differential Expression Among the Three Cell Types

Genes differentially expressed in SLE were likely to be shared between cell types. On average, genes differentially expressed in one cell type were 6.1 times more likely to be differentially expressed toward the same direction and 4.9 times less likely to be differentially expressed toward the opposite direction in the other two cell types. Respectively, 95 and 97 genes had higher and lower expression concordantly in all cell three types. *GTF2E1* (general TF) was the most consistently upregulated gene, increased by 8.5 (B cell), 4.1 (monocyte), and 6.1 (T cell) fold in SLE. *HSPA1A* (the gene encoding the heat shock protein, Hsp70) was 197 (B cell), 33.5 (monocyte), and 17.6 (T cell) fold decreased, making it the most consistently downregulated gene.

**Figure 2:**
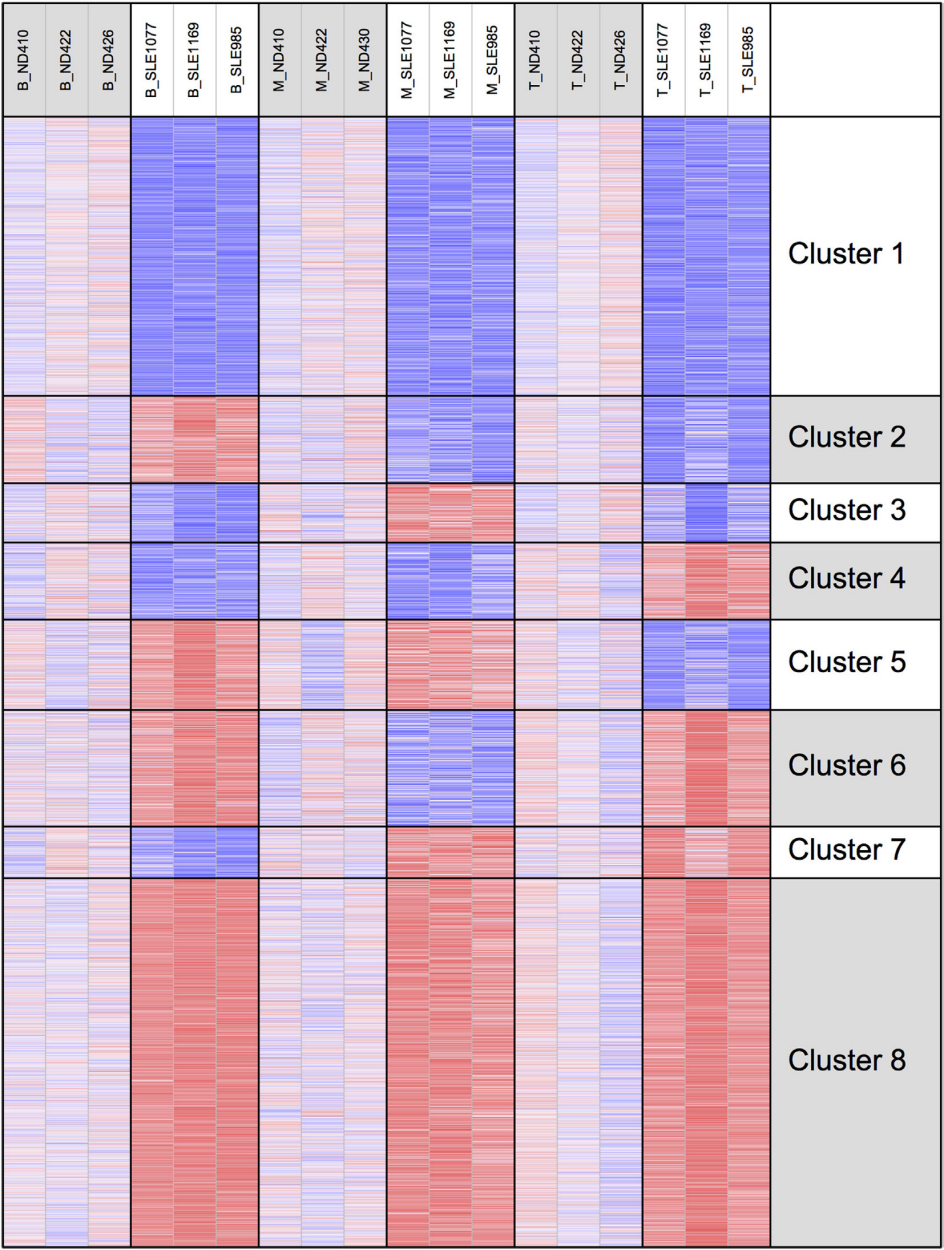
Clustering of genes by gene expression. Cluster analysis was applied to 1,952 genes selected for differential gene expression with *p* < 0.01. Among the eight clusters, the two largest clusters (Clusters 1 and 8) had concordant behavior across all three cell types, accounting for 55% of the differentially expressed genes.

**Figure 3:**
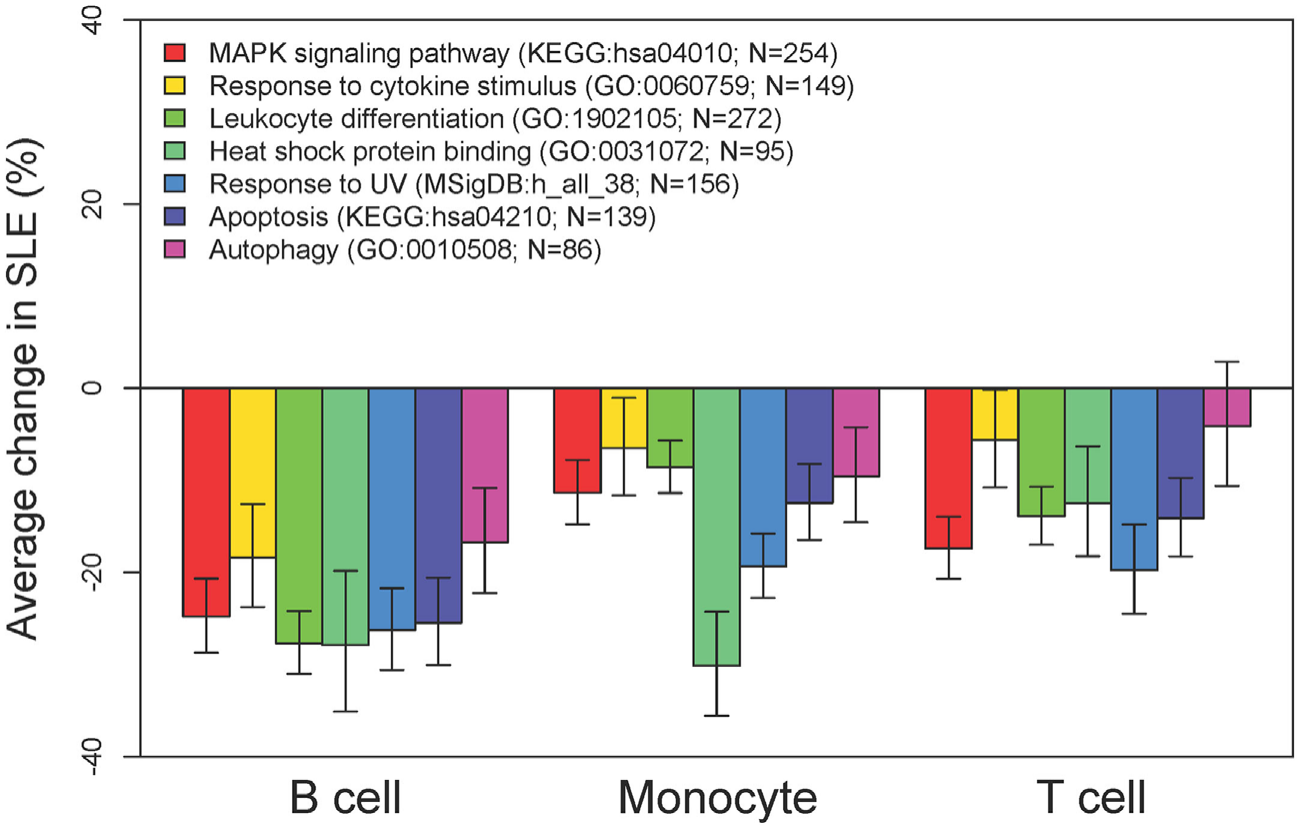
MAP kinases and downstream effects are consistently downregulated. Comparative expression of genes-related MAP kinases and their downstream functions in each of the three cell types. For these categories of downregulated genes, there was high concordance across cell types.

To better examine the concordance and cell-type specific expression, we performed clustering. Clustering was performed across all 18 RNA-seq samples to identify 8 gene clusters having the distinctive co-expression patterns across cell types (Figure 2). The two largest clusters (Cluster 1, downregulated and Cluster 8, upregulated) were concordant across all three types and accounted for 55% of the total differential gene set. One of the gene sets most over-represented in Cluster 1 (downregulated concordantly) was “MAPK signaling pathway.” Genes related to major biological processes regulated by p38 MAP kinases including cytokine response, differentiation, apoptosis, and autophagy, were also downregulated in all three cell types (Figure 3; Table S1 in Supplementary Material). Negative regulators of MAP kinases, *DUSP4/11/12*, all had significantly higher expression in all three cell types. Top gene sets over-represented in Cluster 8 (upregulated concordantly) were related to RNA processing, transport, and translation.

#### Gene Set Analysis

**Figure 4:**
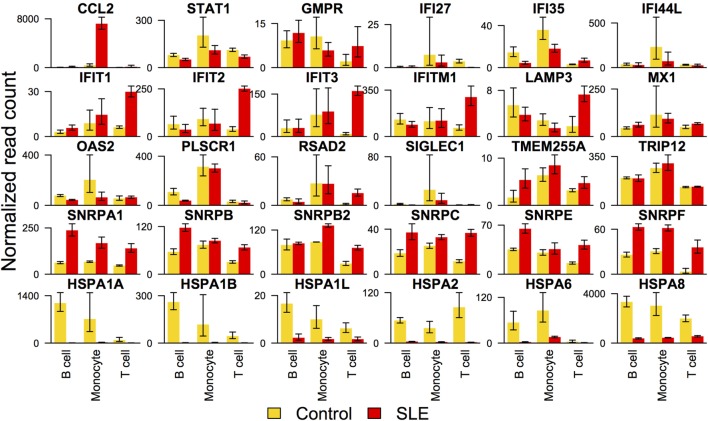
Expression of selected interferon signature genes and heat shock protein genes. Interferon signature genes were not expressed at higher levels in systemic lupus erythematosus (SLE) consistently across all three cell types. In contrast, expression of heat shock proteins was overall downregulated similarly across all three cell types.

We noted that the interferon signal was barely detectable as defined by gene set enrichment. Because it was surprising that the interferon signature was so subtle, we sought gene expression for interferon-responsive genes and tabulated levels in each cell type. When this was performed, we saw distinctive effects in each cell type (Figure 4). In B cells, only *CCL2* was over-expressed in SLE compared with controls with a significant *p*-value. In T cells, *IFIT1, IFIT3, IFITM1, OASL*, and *RSAD2* were over-expressed in SLE compared with controls. In monocytes, *CCL2* and *OASL* were over-expressed in SLE compared with controls. These over-expressed genes were balanced by a group that was under-expressed in SLE compared with controls. In contrast to the interferon response, heat shock proteins were downregulated comparably in all three cell types (Figure 4).

**Figure 5:**
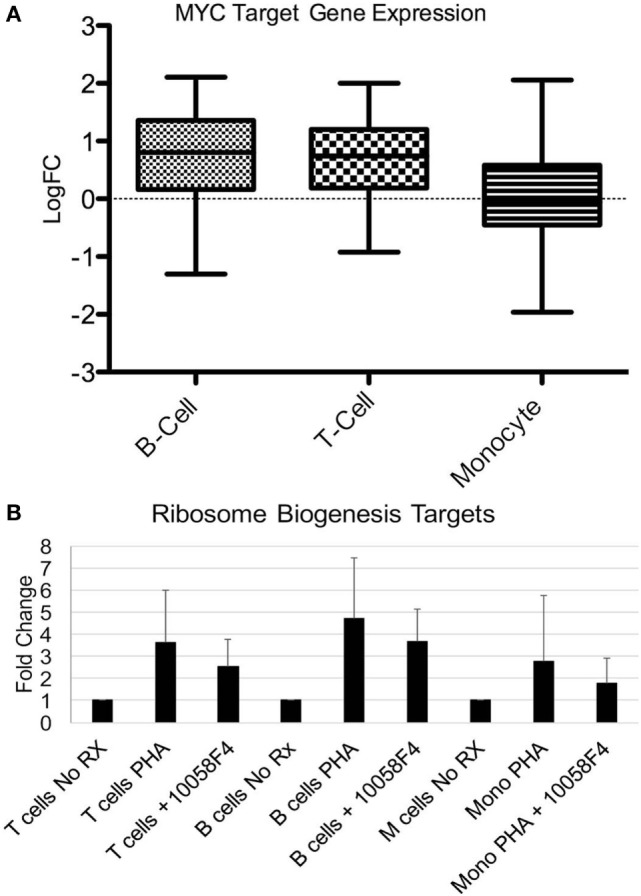
MYC pathway effects on ribosome biogenesis genes. **(A)** Fifty-one MYC targets were manually extracted from the RNA-seq datasets. Expression was expressed as log fold change (Log FC) of systemic lupus erythematosus compared with controls. The expression was averaged. The bars demonstrate mean as a horizontal line and SD as the edges of the bar with the upper and lower bounds denoting maximum and minimum. The increase over the non-MYC targets was significant for T cells (*p* < 0.0001) and B cells (*p* < 0.0001) but not monocytes. **(B)** Six ribosomal biogenesis genes (*THOC1, NUP35, GEMIN2, NUP107, NCBP1*, and *RPP38*) were selected based on level of expression and the presence in all three cell types from the RNA-seq data. Peripheral blood mononuclear cells from healthy donors were stimulated with phytohemagglutinin (PHA) to induce growth. Pre-incubation of the cells with the 100058F4 MYC inhibitor was associated with diminished induction of expression of ribosome biogenesis targets with PHA. Expression of the control gene, *GAPDH*, was not altered by the inhibitor. The decrement related to inhibitor use was significant for T cells (*p* = 1.7 × 10^−5^), B cells (*p* = 8.4 × 10^−7^), and monocytes (*p* = 0.008).

The most consistently upregulated gene set was ribosome biogenesis. Ribosomes increase with proliferation, cell activation, and enlargement. MYC was also a common pathway among upregulated genes in all three cell types (22-fold increased in T cells, 39-fold increased in B cells, and 113-fold increased in monocytes) and MYC is known to regulate ribosome mass and protein synthesis (30). We therefore examined known cell type-independent MYC targets in our gene set (31). MYC targets were significantly increased in T cells and B cells (*p* < 0.0001) although other oncogenes such as *MYB* and *TP53* were not consistently upregulated in our samples (Figure 5A). *MYC* has been previously described as upregulated in SLE and in murine models of SLE (32–34).

To test the relationship between MYC and ribosome biogenesis, PBMC from healthy donors were stimulated with PHA to induce proliferation and the elaboration of cytokines that model immune activation in SLE (Figure 5B). Cells were then separated and qRT-PCR performed for ribosome biogenesis targets that were identified in the RNA-seq data. PHA stimulation increased the ribosome biogenesis targets, as expected. The MYC inhibitor significantly depressed expression of the ribosome biogenesis targets after PHA stimulation in all three cell types (*p* < 0.01). These data support a role for MYC in the expression of ribosome biogenesis genes and provides an important conceptual link between two major gene sets with altered expression in SLE.

### H3K4me3 Analysis

This study was designed to leverage both expression and epigenetics to develop a more complete picture of the pathways dysregulated in SLE and their cell-type concordance. We examined histone marks as a more durable echo of the cells’ exposures and milieu. We employed ChIP-seq for H3K4me3, a mark of active promoters.

**Figure 6:**
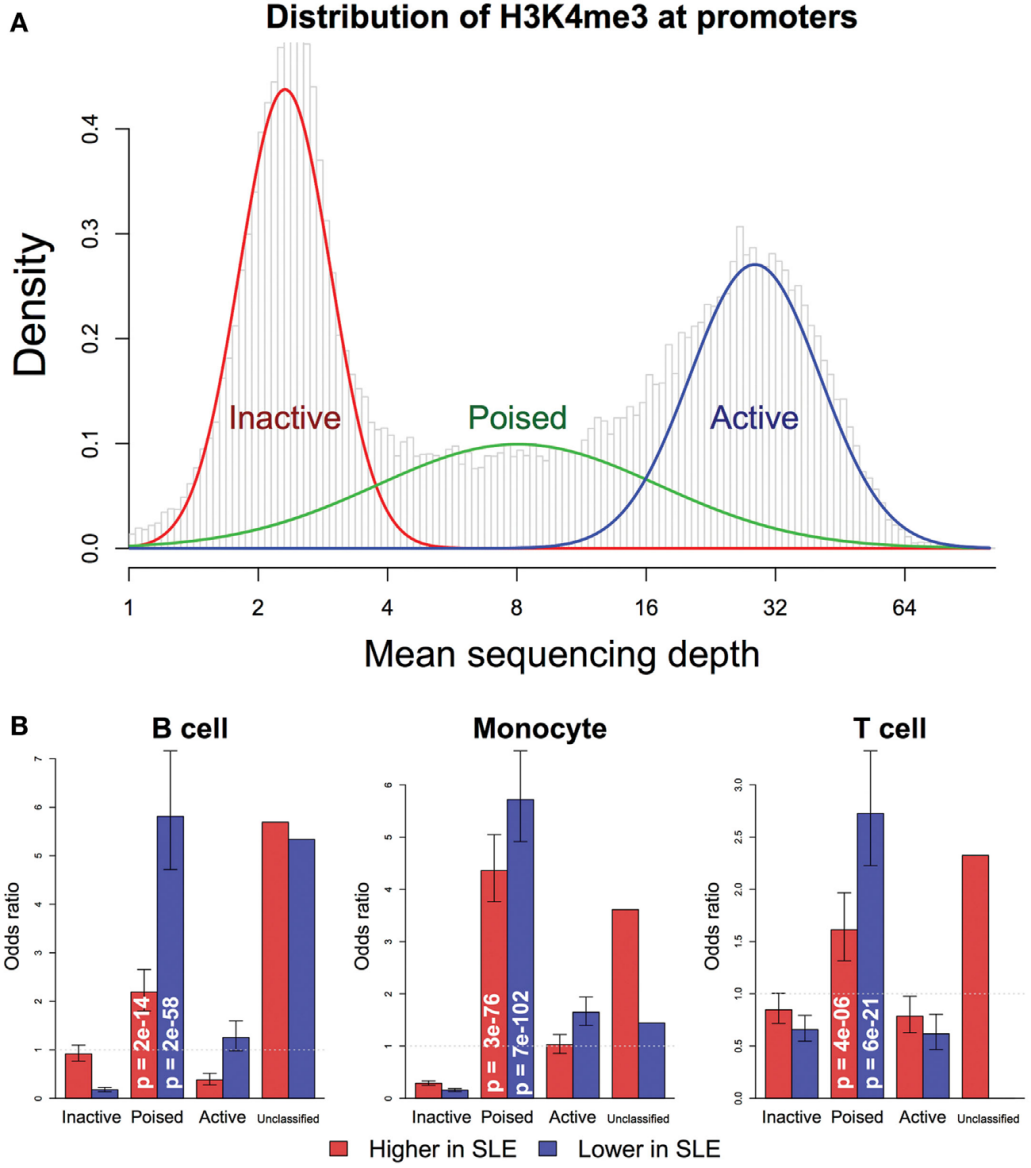
Promoter chromatin affects competence for change in systemic lupus erythematosus (SLE). **(A)** Promoters were classified into three groups: inactive, active, and poised, by fitting their H3K4me3 level of each control sample to the trimodal distribution. Promoters that were not consistently classified into the same group by all samples were put in the unclassified group. **(B)** The likelihood of change in H3K4me3 was associated with control H3K4me3 status, according to Fisher’s exact test. Poised promoters were more likely to exhibit change in SLE, in either direction. *p*-Values are indicated in the bars. The bars indicate odds ratio and SD.

We first classified promoters to analyze changes in SLE based on potential for expression. Based on the average H3K4me3 in controls and the level of expression defined by RNA-seq, promoters were classified into three classes (Figure 6A): (1) The “inactive” promoters had low-H3K4me3 read depth (the left peak), (2) The “active” promoters had high-H3K4me3 read depth (the right peak), and (3) The “poised” promoters had intermediate H3K4me3 read depth. In all three cell types, poised promoters were most likely to have changed H3K4me3 in SLE and none of the promoters changed dramatically from “inactive” to “active” or vice versa (Figure 6B). Comparably to what was seen in the transcriptomic data, more genes had decreased H3K4me3 than increased H3K4me3. These data support a model where cells exhibit altered behavior in SLE but have not been “re-wired” to execute completely novel functions.

#### Differential H3K4me3 in Each Cell Type

Gene set analysis was applied to the change of promoter H3K4me3 in SLE. It identified “cell fate commitment” (GO:0045165) and “cell chemotaxis” (GO:0060326) as the gene sets with the most increased and decreased H3K4me3, respectively, in B cells. In CD3 T cells, MSigDB “TNFA_SIGNALING_VIA_NFKB” pathway was the gene set with the most increased H3K4me3 and “lymphocyte migration” (GO:0072676) was the gene set with the most decreased H3K4me3. We noted that the “TNFA_SIGNALING_VIA_NFKB” pathway was downregulated at the level of RNA and upregulated at the level of promoter H3K4me3 in T cells. This could be interpreted as a chromatin environment favorable for the expression of NFκB targets but lacking the acute stimulation for expression or as a state consistent with endotoxin tolerance. Similarly, in monocytes, “TNFA_SIGNALING_VIA_NFKB” was the gene set with the most H3K4me3 increase, followed by gene sets such as “inflammatory response” (GO:0006954) and “Phagosome” (KEGG: hsa04145).

#### Concordance of H3K4me3 Among the Three Cell Types

H3K4me3 in controls agreed on the classification of over 75% of promoters across the three cell types. Genes with the highest H3K4me3 in a single cell type were associated with cell lineage. For example, genes with the highest promoter H3K4me3 in B cells and T cells were enriched with genes of B cell activation (GO:0042113) and T cell activation (GO:0042110), respectively. Genes with highest H3K4me3 in monocyte were enriched with those related to inflammatory response (GO:0006954) and leukocyte migration (GO:0050900). This analysis demonstrates the fidelity of our ChIP-seq approach by confirming the expected cell type specificity.

There were not many promoters having significant H3K4me3 change in all three cell types, with B cells and monocytes being weakly correlated (correlation coefficient = 0.02). Promoters with increased H3K4me3 in all cell types included *HLAB, RFX2* (regulatory factor X2), and *RARA* (retinoic acid receptor). Promoters with decreased H3K4me3 in all cell types included *KANSL1* (KAT8 regulatory complex), *CX3CR1* (chemokine receptor), and *RORC* (RAR-related orphan receptor).

**Figure 7:**
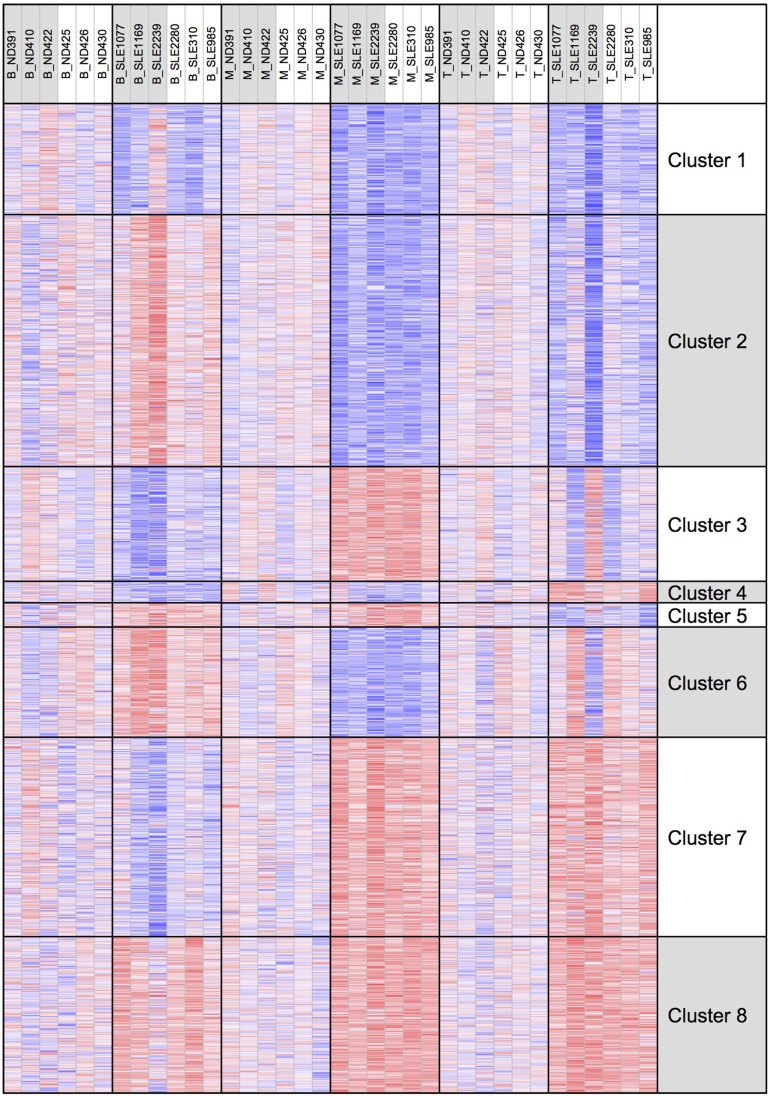
H3K4me3 clustering of the eight gene expression clusters. The eight clusters are displayed with their H3K4me3 peak height.

We examined H3K4me3 using a clustering approach, as was done for the RNA-seq data (Figure 7). Clusters 1 and 8 had decreased and increased H3K4me3, respectively, in all three cell types. The gene set over-represented most significantly in Cluster 1 (downregulated concordantly) was “regulation of leukocyte mediated immunity” (GO:0002703), which includes genes such as *CCR2, IL27RA, STAT5A, STAT6*, and *VAMP2*. The MSigDB “TNFA_SIGNALING_VIA_NFKB” pathway was the gene set most significantly over-represented in Cluster 8 (upregulated concordantly). Clusters 2 and 7 were the two largest clusters, with, respectively, decreased and increased H3K4me3 in both monocyte and T cells. Cluster 2 was over-represented with genes of signaling pathways, such as mTOR, Wnt, and PPAR, and Cluster 7 was most over-represented with DNA repair genes.

#### Concordant Change in Transcription and H3K4me3

**Figure 8:**
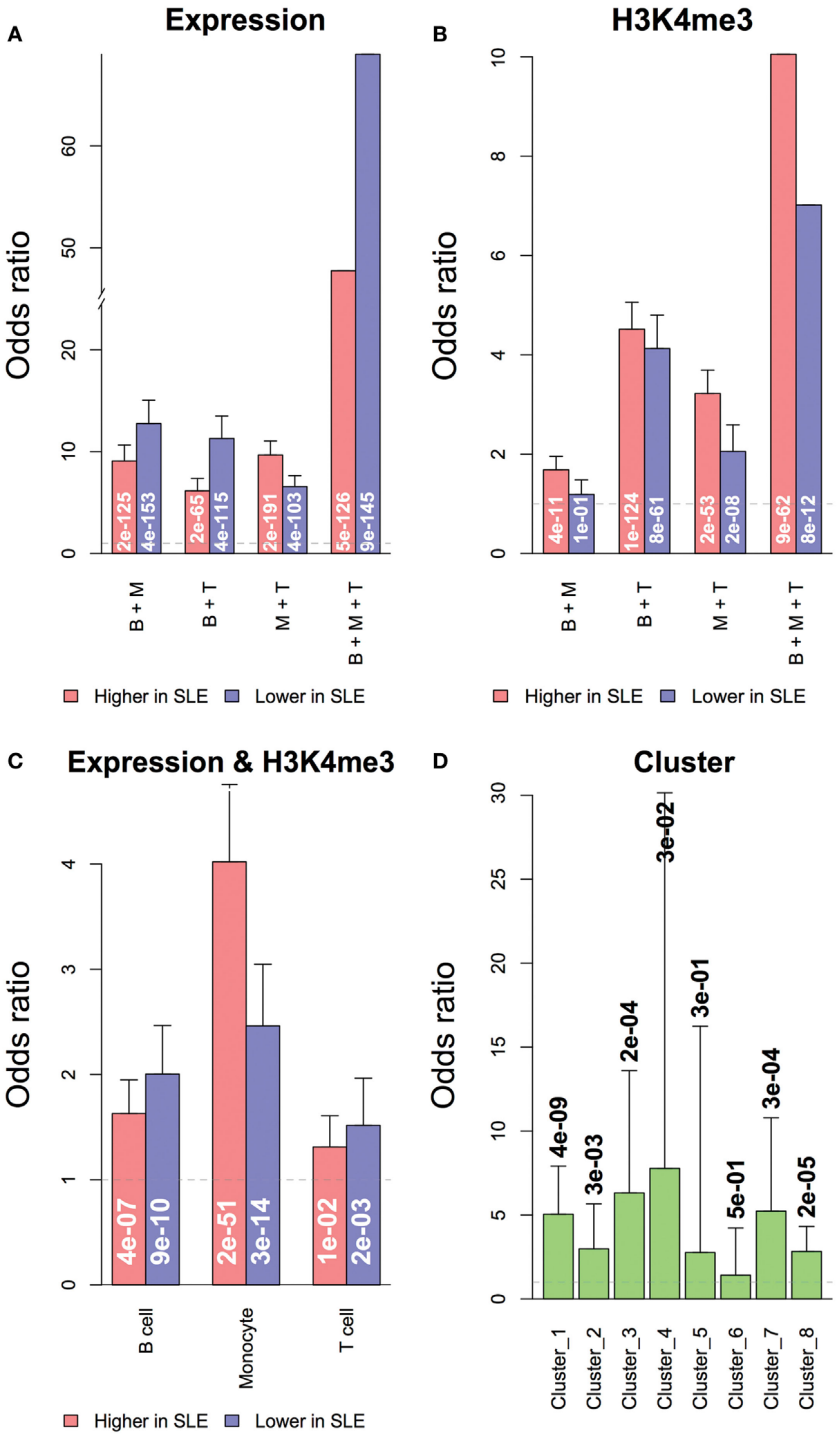
Overlapping of genes with significant differential expression or H3K4me3 in systemic lupus erythematosus (SLE). Genes with differential expression or differential H3K4me3 were likely to be shared across all three cell types. **(A)** Overlapping of genes with differential expression in multiple cell types. **(B)** Overlapping of genes with differential promoter H3K4me3 in multiple cell types. Concordance of expression and H3K4me3 was defined by cell type and cluster. **(C)** Overlapping of genes with differential expression and promoter H3K4me3 in each cell types. **(D)** Overlapping of gene clusters grouped by expression and H3K4me3 data. Odds ratios and labeled *p*-values were calculated by the Exact test.

We asked how much the change of promoter H3K4me3 contributed to the differential expression of genes in SLE. Both RNA and H3K4me3 changes related to SLE were likely to be shared across all three cell types (Figures 8A,B). Genes with significant differential expression were more likely to have significant H3K4me3 change toward the same direction in all three cell types (Figure 8C), and less likely to have H3K4me3 change toward the opposite direction. This is consistent with the role of H3K4me3 in gene regulation. The gene clusters defined by transcription patterns were also significantly overlapped to their counterparts defined by H3K4me3 patterns (Figure 8D). However, less than 10% of all genes with significant differential expression in SLE also had significant differential H3K4me3 toward the same direction, suggesting that the differential expression of most genes was not directly associated with H3K4me3 change, consistent with the role of H3K4me3 in setting the potential for expression. Noticeably, more genes had concordant transcription and H3K4me3 changes in monocytes than in the other two cell types. The top genes having a significant increase of both in monocytes were enriched with genes related to cell chemotaxis, including *CCL2, CCL7, CCR1, CXCR1, IL1R1*, and *TRIM1*.

#### Concordant Change in Gene Sets Defined by Transcription and H3K4me3

Our goal was to leverage this robust data set with both chromatin and RNA level results to understand commonalities and differences between cell effects in SLE. We analyzed themes related to cell biological processes since individual genes and promoters might be differentially regulated in the three cell types. Based on gene set analysis, genes related to RNA transport (KEGG:hsa03013) and leukocyte activation (GO:0045321) had increased and decreased transcription and H3K4me3, respectively, in all three cell types. 57% of the RNA transport genes with increased transcription in B cells also had increased promoter H3K4me3. Table S2 in Supplementary Material lists additional gene sets with concordant and discordant transcription and H3K4me3 changes.

**Figure 9:**
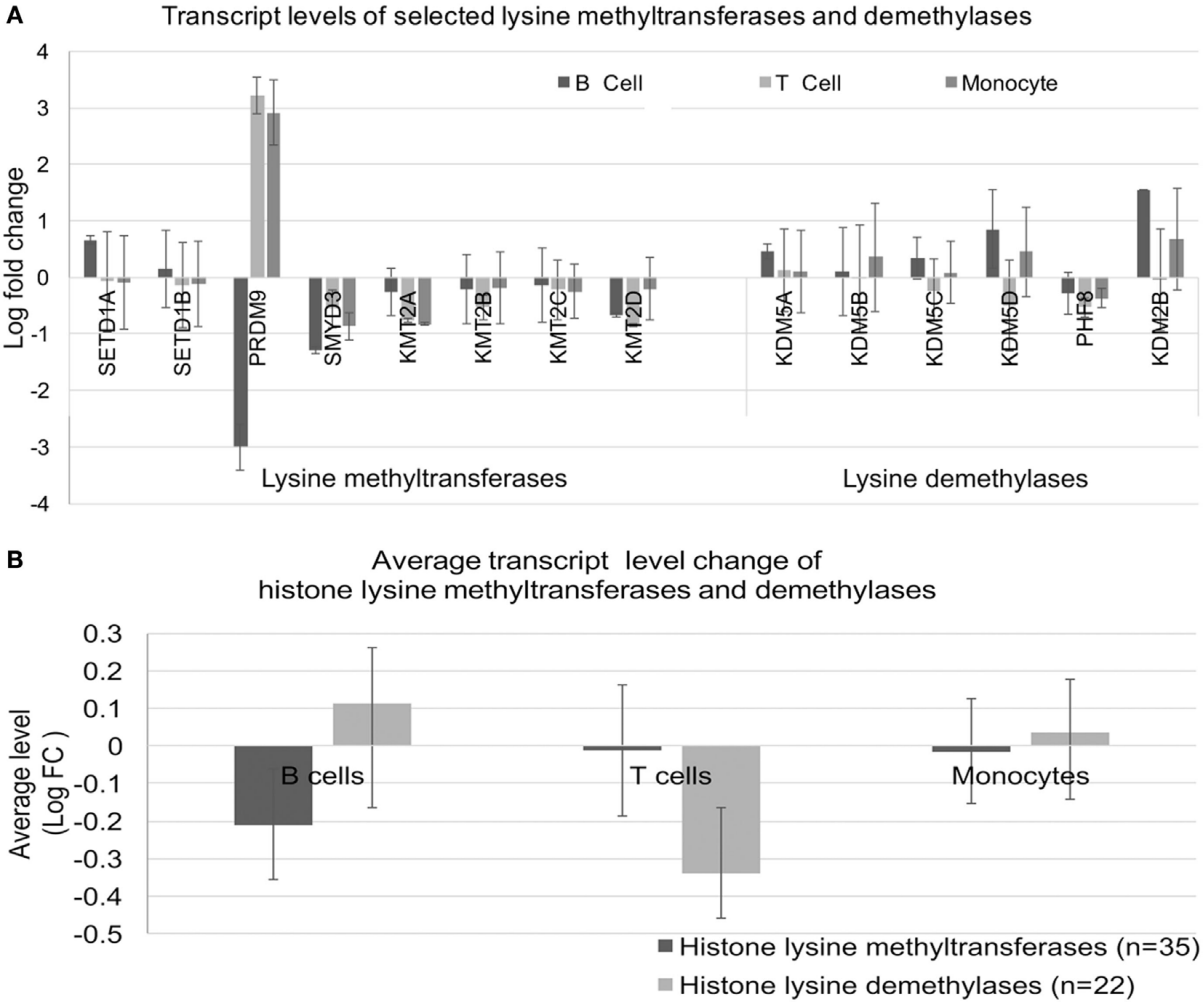
Expression of lysine methyltransferase and lysine demethylase enzymes. **(A)** Expression of a selected set of enzymes was analyzed in the three cell types. **(B)** The expression levels were averaged for the sets defined in **(A)** to identify class-specific effects.

We considered whether the effects on histone methylation could reflect dysregulated expression of histone methyltransferases or demethylase enzymes. To this end, we examined a select set of histone methyltransferases and demethylases (Figure 9A). The expression of PRDM9 was the most dysregulated in SLE; however, alterations were not consistent across cell types. When we examined the overall levels of transcripts for the three cell types using the entire set of enzymes, there was no clear imbalance in transcription of methyltransferases or demethylases (Figure 9B).

Our analysis also identified region-specific effects. There were genomic regions within which genes had concordant transcription and promoter H3K4me3 changes. For example, a ~300 kb region on chromosome 17 had decreased RNA and H3K4me3 in B cells from SLE patients. The *CD300* gene family resides in this region. In SLE monocytes, there was a decrease in both transcription and H3K4me3 within a ~400 kb region on chromosome 7, a region that includes members of *GIMAP* gene family. *GIMAP* genes are involved in lymphocyte development. Gene variants of *GIMAP* family members have been associated with atopic and autoimmune disease (35).

#### TF Motif Analysis

**Figure 10:**
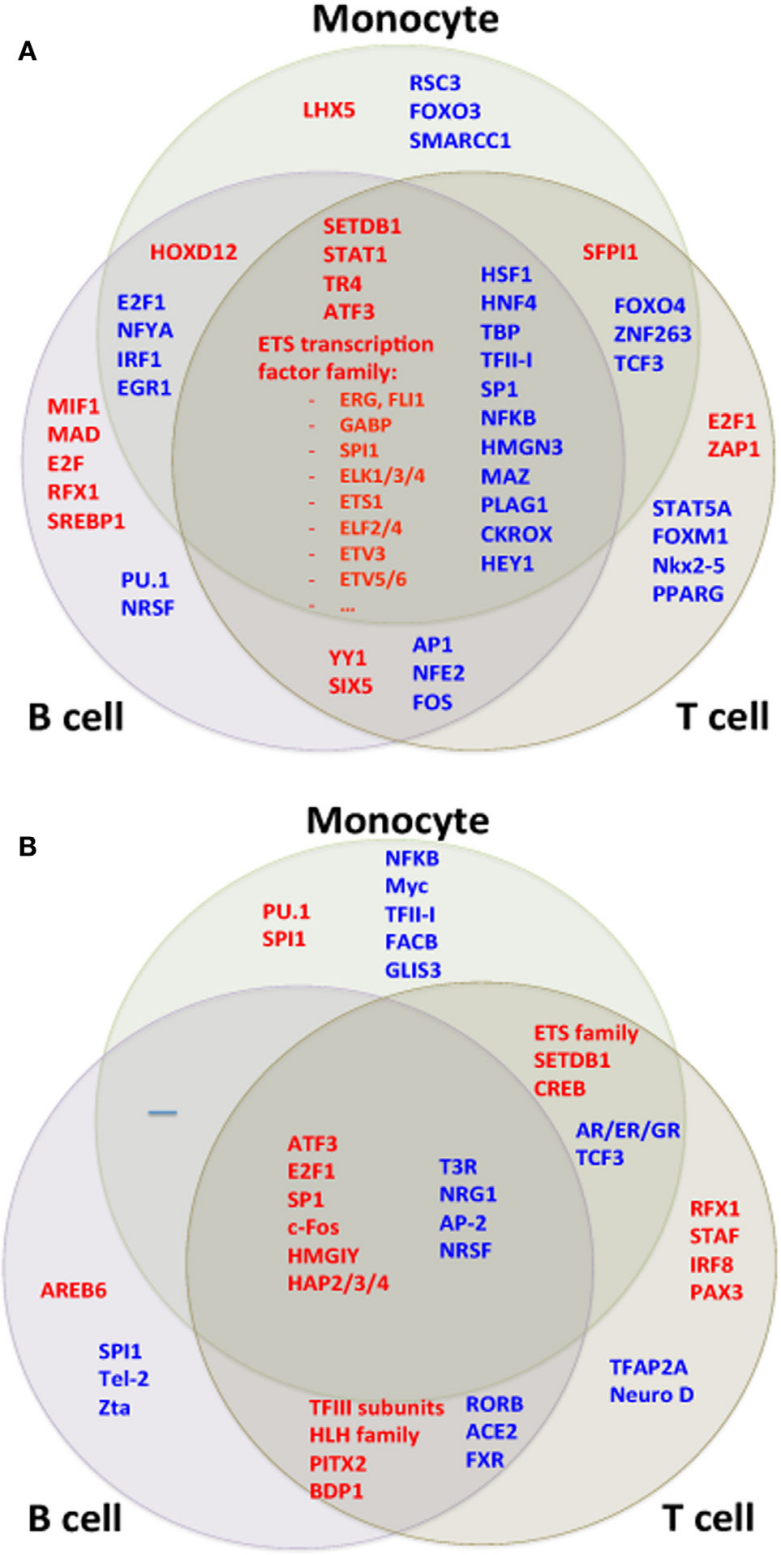
Transcription factor (TF) motifs according to cell type. TF motifs were analyzed by position weight matrix. TF motifs with the most significant change in **(A)** expression and **(B)** H3K4me3 were both shared and unique to cell types.

In addition to analyzing gene sets and region effects, we analyzed upstream transcriptional activators, reasoning that the individual genes induced or repressed in the SLE disease process may differ from cell type to cell type but that there might be common signaling pathways and TFs amenable to drug targeting. Gene set analysis of TF targets revealed strong agreement between the cell types using the RNA-seq dataset. Most noticeably, many members of the ETS (E26 transformation specific) TF family had higher target expression in all cell types (Figure 10A).

**Figure 11:**
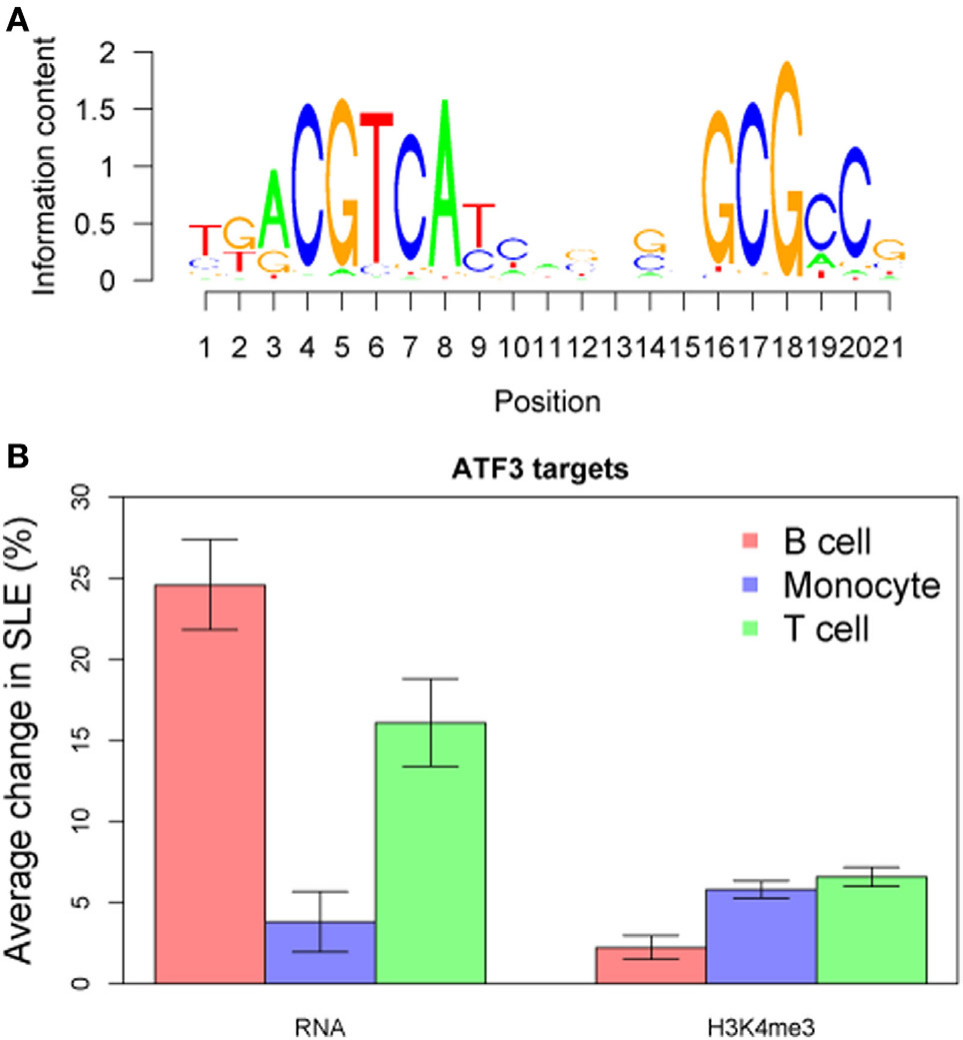
ATF3 was identified as a central transcription factor concordant across all three cell types and both RNA-seq and chromatin immunoprecipitation-seq. **(A)** The ATF3 position weight matrix binding site is displayed. **(B)** The ATF3 target genes are overall increased for both expression and H3K4me3 peak height.

We compared TF targets using the ChIP-seq dataset as well after adjusting data for the GC content of their promoter sequences. Target sets of TFs such as SP1, ATF3, and HMGA1 had increased H3K4me3 in all cell types (Figure 10B). We wished to determine if there was a common pathway linking H3K4me3 and gene expression. ATF3 was identified as statistically associated across all cell types in both RNA-seq and ChIP-seq data sets (Figure 11). *CDK11A*, a kinase gene involved in apoptosis, is a target of ATF3 and had at least 60% increase of both expression and H3K4me3 in all three cell types, supporting a functional role for the ATF3 identified by the *in silico* approach.

### Genome-Wide Association-Identified Single Nucleotide Polymorphisms Associated With SLE Analyzed

**Figure 12:**
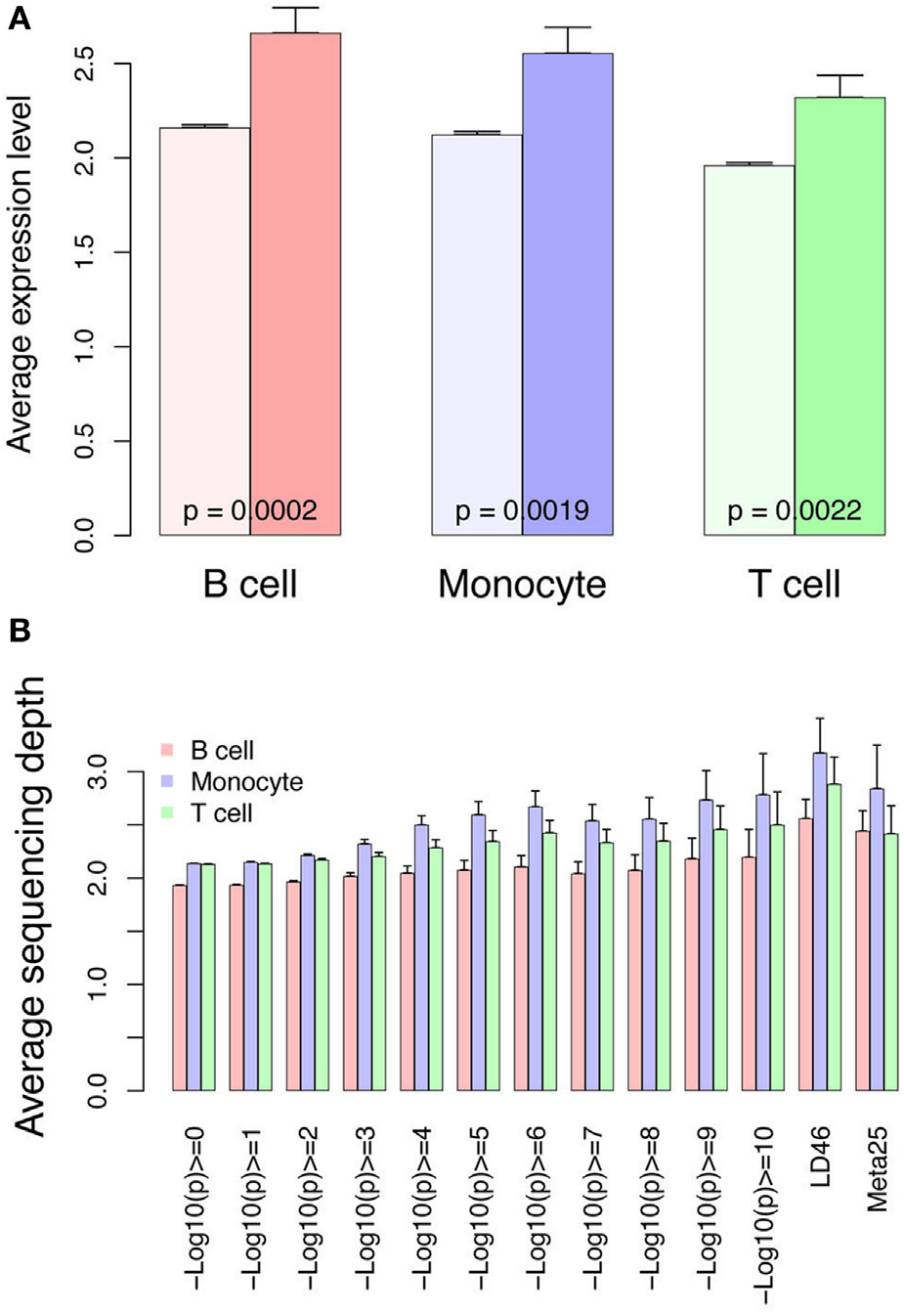
RNA and H3K4me3 depth at regions surrounding genome-wide association study variants. **(A)** RNA abundance at the TSS nearest the 143 SNPs curated from three studies and meta-analyses. This graph portrays the RNA at the TSS nearest the 143 SNPs (dark colors) compared with all other genes in the control samples (light bars). **(B)** H3K4me3 sequencing depth at the systemic lupus erythematosus (SLE) SNPs. This graph portrays all SLE SNPs binned according to *p*-value of SNP association (*x*-axis). The LD46 and META25 SNP sets represent top SNP sets from manuscripts (see Materials and Methods). The bars indicate sequencing depth in the controls at the three different cell types.

In addition to gene expression studies identifying the interferon signature, GWAS has been instrumental in identifying pivotal pathways in SLE such as immune complex processing, type I interferons, and lymphocyte signaling (1, 36). Some identified variants have been analyzed with respect to functional differences, however, most GWAS variants are found in regulatory regions not coding regions, thus hampering functional analysis. Additionally, cell-specific effects may complicate efforts to define functional consequences of variants. We therefore cross-referenced our RNA-seq and ChIP-seq data sets with published GWAS data using three sets of variants. We first hypothesized that the GWAS variants would be enriched at locations with higher RNA expression. This should be reflected in higher RNA and H3K4me3 signals near the variants. We compared expression from control cells near the combined set of 143 GWAS variants to all other genes (Figure 12A). All three cell types have higher expression from the TSS nearest the GWAS variants, supporting a functional role for the variants. Similarly, H3K4me3 was higher surrounding the GWAS variants with the highest *p*-values for SLE association (displayed on the *X*-axis) and the two curated sets of variants, LD46 and Meta25, again supporting functionality (Figure 12B).

**Figure 13:**
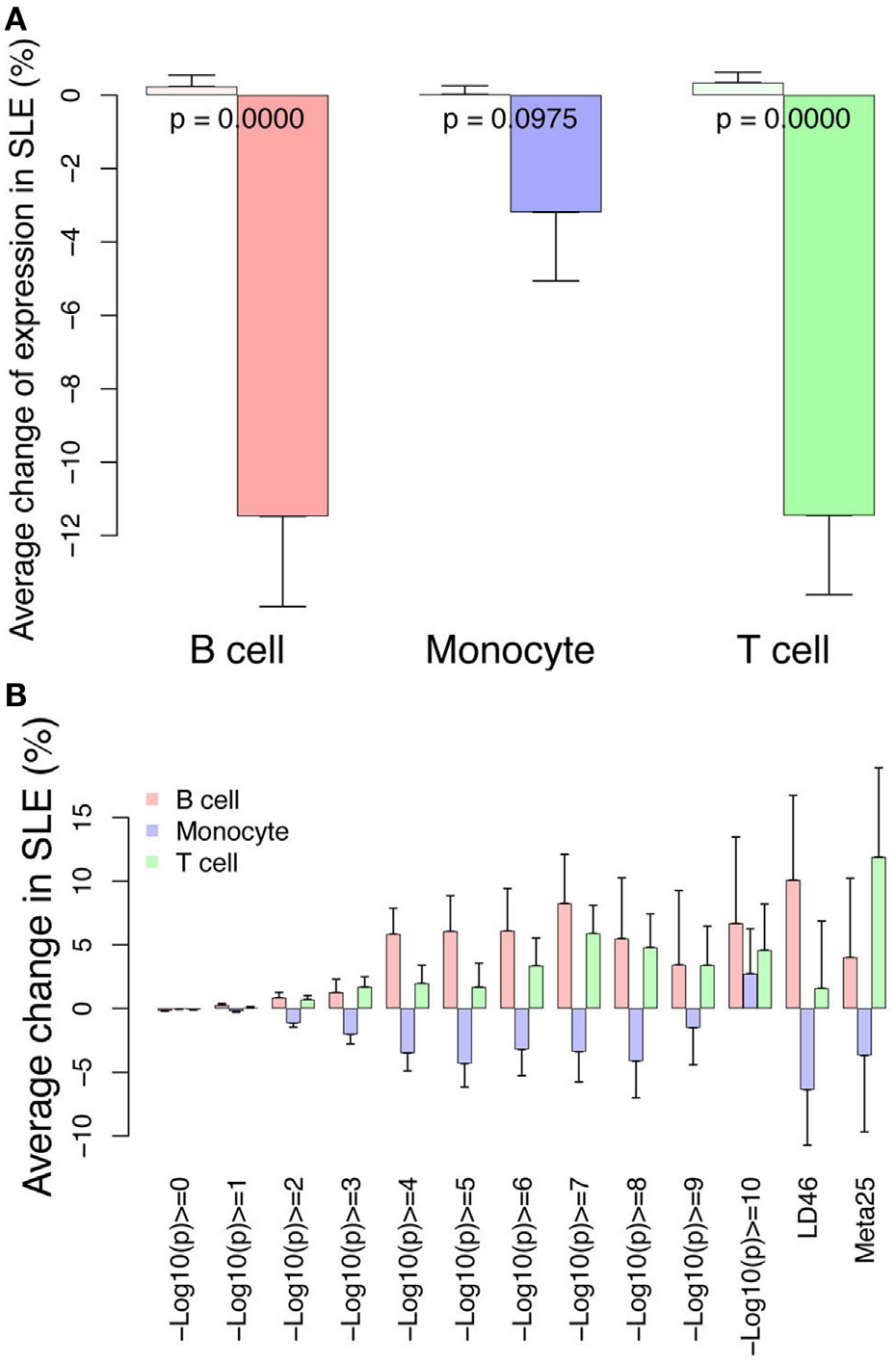
Differential RNA and H3K4me3 depth at regions surrounding genome-wide association study (GWAS) variants. **(A)** Changes in RNA abundance related to systemic lupus erythematosus (SLE) at the TSS nearest to the 143 GWAS SNPs. There is overall decreased expression. Dark bars indicate GWAS SNP-associated RNAs and light bars indicate all others. *p*-Values are listed across the bars. **(B)** Changes in H3K4me3 at the 143 GWAS SNPs binned according to *p*-value of SNP association (*x*-axis). The LD46 and META25 SNP sets represent top SNP sets from manuscripts (see Materials and Methods). The bars indicate change in SLE compared with control.

We further reasoned that SLE-associated genomic variants might be further enriched with changes in expression related to SLE or changed H3K4me3 related to SLE. We therefore looked at change in expression and change in H3K4me3 near the GWAS variants. Near the GWAS variants, there was an impressive decrease in RNA abundance in all three cell types (Figure 13A). The change in H3K4me3 near GWAS variants was similarly analyzed and monocytes exhibited decreased H3K4me3 while T cells and B cells exhibited an increase in H3K4me3 near GWAS variants (Figure 13B). The SNPs with the highest *p*-value (displayed on the *X*-axis) have the strongest association. These data are consistent in supporting the concept that GWAS variants impact gene regulation.

## Discussion

To understand whether conserved pathways affect multiple cell types in SLE, we performed a detailed analysis of RNA and H3K4me3 changes in SLE. We selected patients with low-disease activity to focus on core facets of the SLE transcriptome and to avoid medication effects. 55% of the genes with altered expression were concordant across the three cell types. The most pivotal modules of gene expression identified in all three cell types in our study were completely fundamental to cell biology. RNA-seq analysis identified MAP kinase and NFκB pathways as strongly downregulated in SLE. Diminished MAP kinases have been previously described in SLE T cells (37, 38). We also identified ribosome biogenesis as a concordantly upregulated gene set and linked it to the MYC pathway. Increased *MYC* expression has been seen in SLE and is known to upregulate ribosome biogenesis (30, 32–34, 39). MAP kinases promote apoptosis *via* MYC (40–42). Thus, the lower levels of MAP kinase transcripts in SLE cells would be predicted to have the effect of promoting cell survival, growth, and proliferation, all settings where ribosome biogenesis is increased (Figure S1 in Supplementary Material). A curious finding was enrichment of NFκB-regulated genes among downregulated RNAs but increased H3K4me3. NFκB-dependent transcriptional activation relies on H3K4 methyltransferases such as MLL and SET families recruited by MKL1 (43). Thus, the pattern we identified is most consistent with a state of endotoxin tolerance (44).

Our ultimate goal was to define pivotal modulators of gene expression. We therefore examined upstream TF motifs and pathways using an *in silico* approach. The TFs implicated in concordant transcription changes in all three cell types were ETS family members and STAT1, among others. These two TFs have been highlighted because of their recognized role in SLE (45–47). ATF3 was notable because it reached significance when analyzed across all cell types and in both RNA-seq and ChIP-seq data sets and ATF3 targets exhibited increased expression. ATF3 is inducible by type I interferons in macrophages, providing a potential mechanism for increased function in SLE (48–50). ATF3 has been ascribed roles as diverse as tumor suppression, development, and cell survival, however, little is known regarding its role specifically in SLE.

We found a limited signal of type I interferon effects with diverse genes impacted across the three cell types. The type I interferon signature appears to be less robust in purified cell populations studied by other groups as well (9, 10). In our study, this could be due to our focus on patients with low-disease activity, ascertainment of patients with autoantibody profiles that are less associated with type I interferon signatures (51, 52), or an effect related the separation of cell populations and removal of low-density neutrophils.

We analyzed our data from the perspective of known variants associated with SLE because they have been postulated to act primarily at the level of gene regulation. Cross-referencing of SNP loci with DNase hypersensitive sites or other chromatin marks have generally utilized existing datasets from normal tissues (53, 54). These data have implicated T cells (CD4 > CD8), B cells, and monocytes in SLE. One study utilizing neutrophil ChIP-seq data found enrichment of genes with potential ETS TF binding near SLE GWAS SNPs (27). To our knowledge, there are no previous studies that have used disease-related changes in RNA and H3K4me3 to analyze GWAS variants. This study offers a new perspective on the function of the GWAS variants using our unique lens of SLE-related changes to the transcriptome and epigenome. This paves the way for improved approaches to functionality.

Our study had several limitations including a limited sample size, a result of pairing RNA, and ChIP-seq in three cell types. We also chose to focus on a single histone modification. We had previously examined H4 acetylation and H3K4me3 (18, 20, 21). This study focused on H3K4me3 due to the greater understanding of the variables impacting this specific modification. Studies of other modifications, other cell types, and validating patient cohorts will improve our overall understanding of the epigenome of SLE. Our GWAS analysis focused on the region immediately surrounding the variant. SNP linkage disequilibrium blocks may be much larger and therefore the analysis may have under-estimated the effect. Nevertheless, our study provides unique new data on the pathogenesis of SLE using an innovative approach. This study is the first to cross-reference GWAS data with chromatin marks directly impacted by disease. This study presents the most robust analysis of gene expression effects in purified cell types to begin to dissect the concordant and discordant effects in disease. A pivotal and surprising finding is that RNAs cross-referenced with GWAS SNPs had markedly decreased expression in SLE. This study also identified potential drug targets in ETS family members and ATF3. ETS family TFs have been implicated in SLE (45, 55, 56) and represent a credible target for drug development.

In summary, this study provides key insights into molecular pathways relevant in SLE. Slightly more than half of the gene expression changes in SLE patients were concordant. This approach could facilitate efforts at drug discovery where pathologic cell behavior could be more effectively targeted by focusing on concordant pathways. Nevertheless, this study also provides a sobering view of the complexity of this disease. Nearly, half of the gene expression changes were cell specific and identifying the key mediators in distinct cells may prove to be complex. As a first analysis of combined gene expression and histone modifications in SLE across different cell types, this study provides both hope and reservation. This innovative approach to focus GWAS data and to define conserved pathways offers strategies for drug development and analysis of variants.

## Ethics Statement

The female healthy donor blood samples were obtained from the Center for AIDS Research, under a protocol approved by the University of Pennsylvania Perelman School of Medicine Institutional Review Board (IRB) in accordance with their guidelines. All subjects gave informed consent in accordance with the Declaration of Helsinki. Samples were handled identically as the shipped SLE samples. The female SLE patient blood samples were from the Johns Hopkins Lupus Cohort (23, 24) under a separate IRB protocol in accordance with the recommendations and guidelines of the Johns Hopkins IRB Committee. All patients gave informed consent in accordance with the Declaration of Helsinki.

## Author Contributions

LHS, LS, and KM performed experiments and did data analysis. ZZ, KS, and MP did data analysis and wrote the manuscript.

## Conflict of Interest Statement

The authors declare that the research was conducted in the absence of any commercial or financial relationships that could be construed as a potential conflict of interest.

## References

[1] Tsokos GC Lo MS Costa Reis P Sullivan KE. New insights into the immunopathogenesis of systemic lupus erythematosus. Nat Rev Rheumatol (2016) 12(12):716–30. 10.1038/nrrheum.2016.186 27872476

[2] Baechler EC Batliwalla FM Karypis G Gaffney PM Ortmann WA Espe KJ Interferon-inducible gene expression signature in peripheral blood cells of patients with severe lupus. Proc Natl Acad Sci U S A (2003) 100(5):2610–5. 10.1073/pnas.0337679100 12604793 PMC151388

[3] Bennett L Palucka AK Arce E Cantrell V Borvak J Banchereau J Interferon and granulopoiesis signatures in systemic lupus erythematosus blood. J Exp Med (2003) 197(6):711–23. 10.1084/jem.20021553 12642603 PMC2193846

[4] Kirou KA Lee C George S Louca K Papagiannis IG Peterson MG Coordinate overexpression of interferon-alpha-induced genes in systemic lupus erythematosus. Arthritis Rheum (2004) 50(12):3958–67. 10.1002/art.20798 15593221

[5] Baechler EC Bauer JW Slattery CA Ortmann WA Espe KJ Novitzke J An interferon signature in the peripheral blood of dermatomyositis patients is associated with disease activity. Mol Med (2007) 13(1–2):59–68. 10.2119/2006-00085.Baechler 17515957 PMC1869622

[6] Mavragani CP Sagalovskiy I Guo Q Nezos A Kapsogeorgou EK Lu P Expression of long interspersed nuclear element 1 retroelements and induction of type I interferon in patients with systemic autoimmune disease. Arthritis Rheumatol (2016) 68(11):2686–96. 10.1002/art.39795 27338297 PMC5083133

[7] Lubbers J Brink M van de Stadt LA Vosslamber S Wesseling JG van Schaardenburg D The type I IFN signature as a biomarker of preclinical rheumatoid arthritis. Ann Rheum Dis (2013) 72(5):776–80. 10.1136/annrheumdis-2012-202753 23434571

[8] Shi L Zhang Z Yu AM Wang W Wei Z Akhter E The SLE transcriptome exhibits evidence of chronic endotoxin exposure and has widespread dysregulation of non-coding and coding RNAs. PLoS One (2014) 9(5):e93846. 10.1371/journal.pone.0093846 24796678 PMC4010412

[9] Kyogoku C Smiljanovic B Grun JR Biesen R Schulte-Wrede U Haupl T Cell-specific type I IFN signatures in autoimmunity and viral infection: what makes the difference? PLoS One (2013) 8(12):e83776. 10.1371/journal.pone.0083776 24391825 PMC3877094

[10] Sharma S Jin Z Rosenzweig E Rao S Ko K Niewold TB. Widely divergent transcriptional patterns between SLE patients of different ancestral backgrounds in sorted immune cell populations. J Autoimmun (2015) 60:51–8. 10.1016/j.jaut.2015.04.002 25921064 PMC4457613

[11] Olferiev M Jacek E Kirou KA Crow MK. Novel molecular signatures in mononuclear cell populations from patients with systemic lupus erythematosus. Clin Immunol (2016) 172:34–43. 10.1016/j.clim.2016.08.018 27576056

[12] Dozmorov MG Dominguez N Bean K Macwana SR Roberts V Glass E B-cell and monocyte contribution to systemic lupus erythematosus identified by cell-type-specific differential expression analysis in RNA-seq data. Bioinform Biol Insights (2015) 9(Suppl 3):11–9. 10.4137/BBI.S29470 26512198 PMC4599594

[13] Li Y Gorelik G Strickland FM Richardson BC. Oxidative stress, T cell DNA methylation, and lupus. Arthritis Rheumatol (2014) 66(6):1574–82. 10.1002/art.38427 24577881 PMC4141415

[14] Yung R Powers D Johnson K Amento E Carr D Laing T Mechanisms of drug-induced lupus. II. T cells overexpressing lymphocyte function-associated antigen 1 become autoreactive and cause a lupuslike disease in syngeneic mice. J Clin Invest (1996) 97(12):2866–71. 10.1172/JCI118743 8675699 PMC507381

[15] Sawalha AH Wang L Nadig A Somers EC McCune WJ Michigan Lupus C Sex-specific differences in the relationship between genetic susceptibility, T cell DNA demethylation and lupus flare severity. J Autoimmun (2012) 38(2–3):J216–22. 10.1016/j.jaut.2011.11.008 22305513 PMC3313010

[16] Javierre BM Fernandez AF Richter J Al-Shahrour F Martin-Subero JI Rodriguez-Ubreva J Changes in the pattern of DNA methylation associate with twin discordance in systemic lupus erythematosus. Genome Res (2010) 20(2):170–9. 10.1101/gr.100289.109 20028698 PMC2813473

[17] Zhang Z Shi L Song L Ephrem E Petri M Sullivan KE. Interferon regulatory factor 1 marks activated genes and can induce target gene expression in systemic lupus erythematosus. Arthritis Rheumatol (2015) 67(3):785–96. 10.1002/art.38964 25418955 PMC4342285

[18] Zhang Z Song L Maurer K Petri MA Sullivan KE. Global H4 acetylation analysis by ChIP-chip in systemic lupus erythematosus monocytes. Genes Immun (2010) 11(2):124–33. 10.1038/gene.2009.66 19710693 PMC2832080

[19] Shi L Perin JC Leipzig J Zhang Z Sullivan KE. Genome-wide analysis of interferon regulatory factor I binding in primary human monocytes. Gene (2011) 487(1):21–8. 10.1016/j.gene.2011.07.004 21803131 PMC3167955

[20] Shi L Zhang Z Song L Leung YT Petri MA Sullivan KE. Monocyte enhancers are highly altered in systemic lupus erythematosus. Epigenomics (2015) 7(6):921–35. 10.2217/epi.15.47 26442457 PMC4864065

[21] Zhang Z Shi L Dawany N Kelsen J Petri MA Sullivan KE. H3K4 tri-methylation breadth at transcription start sites impacts the transcriptome of systemic lupus erythematosus. Clin Epigenetics (2016) 8:14. 10.1186/s13148-016-0179-4 26839600 PMC4736279

[22] Scharer CD Blalock EL Barwick BG Haines RR Wei C Sanz I ATAC-seq on biobanked specimens defines a unique chromatin accessibility structure in naive SLE B cells. Sci Rep (2016) 6:27030 10.1038/srep27030 27249108 PMC4888756

[23] Fangtham M Petri M. 2013 update: Hopkins lupus cohort. Curr Rheumatol Rep (2013) 15(9):360. 10.1007/s11926-013-0360-0 23888367 PMC3756858

[24] Petri M. Hopkins lupus cohort. 1999 update. Rheum Dis Clin North Am (2000) 26:199–213. 10.1016/S0889-857X(05)70135-6 10768209

[25] Garrett S Dietzmann-Maurer K Song L Sullivan KE. Polarization of primary human monocytes by IFN-gamma induces chromatin changes and recruits RNA Pol II to the TNF-alpha promoter. J Immunol (2008) 180(8):5257–66. 10.4049/jimmunol.180.8.5257 18390706

[26] Hom G Graham RR Modrek B Taylor KE Ortmann W Garnier S Association of systemic lupus erythematosus with C8orf13-BLK and ITGAM-ITGAX. N Engl J Med (2008) 358(9):900–9. 10.1056/NEJMoa0707865 18204098

[27] Hui-Yuen JS Zhu L Wong LP Jiang K Chen Y Liu T Chromatin landscapes and genetic risk in systemic lupus. Arthritis Res Ther (2016) 18(1):281. 10.1186/s13075-016-1169-9 27906046 PMC5134118

[28] Bentham J Morris DL Graham DSC Pinder CL Tombleson P Behrens TW Genetic association analyses implicate aberrant regulation of innate and adaptive immunity genes in the pathogenesis of systemic lupus erythematosus. Nat Genet (2015) 47(12):1457–64. 10.1038/ng.3434 26502338 PMC4668589

[29] Sun C Molineros JE Looger LL Zhou XJ Kim K Okada Y High-density genotyping of immune-related loci identifies new SLE risk variants in individuals with Asian ancestry. Nat Genet (2016) 48(3):323–30. 10.1038/ng.3496 26808113 PMC4767573

[30] van Riggelen J Yetil A Felsher DW MYC as a regulator of ribosome biogenesis and protein synthesis. Nat Rev Cancer (2010) 10(4):301–9. 10.1038/nrc2819 20332779

[31] Ji H Wu G Zhan X Nolan A Koh C De Marzo A Cell-type independent MYC target genes reveal a primordial signature involved in biomass accumulation. PLoS One (2011) 6(10):e26057. 10.1371/journal.pone.0026057 22039435 PMC3198433

[32] Deguchi Y Hara H Negoro S Kakunaga T Kishimoto S. Protooncogene expression in peripheral blood mononuclear cells from patients with systemic lupus erythematosus as an indicator of the disease activity. Clin Immunol Immunopathol (1987) 45(3):424–39. 10.1016/0090-1229(87)90094-8 3677489

[33] Eleftheriades EG Boumpas DT Balow JE Tsokos GC. Transcriptional and post-transcriptional mechanisms are responsible for the increased expression of c-myc protooncogene in lymphocytes from patients with systemic lupus erythematosus. Clin Immunol Immunopathol (1989) 52(3):507–15. 10.1016/0090-1229(89)90163-3 2474397

[34] Kitajima T Furukawa F Kanauchi H Imamura S Ogawa K Sugiyama T. Histological detection of c-myb and c-myc proto-oncogene expression in infiltrating cells in cutaneous lupus erythematosus-like lesions of MRL/l mice by in situ hybridization. Clin Immunol Immunopathol (1992) 62(1 Pt 1):119–23. 10.1016/0090-1229(92)90031-I 1728975

[35] Heinonen MT Laine AP Soderhall C Gruzieva O Rautio S Melen E GIMAP GTPase family genes: potential modifiers in autoimmune diabetes, asthma, and allergy. J Immunol (2015) 194(12):5885–94. 10.4049/jimmunol.1500016 25964488 PMC4456634

[36] Harley IT Kaufman KM Langefeld CD Harley JB Kelly JA. Genetic susceptibility to SLE: new insights from fine mapping and genome-wide association studies. Nat Rev Genet (2009) 10(5):285–90. 10.1038/nrg2571 19337289 PMC2737697

[37] Gorelik G Fang JY Wu A Sawalha AH Richardson B. Impaired T cell protein kinase C delta activation decreases ERK pathway signaling in idiopathic and hydralazine-induced lupus. J Immunol (2007) 179(8):5553–63. 10.4049/jimmunol.179.8.5553 17911642

[38] Yasuda S Stevens RL Terada T Takeda M Hashimoto T Fukae J Defective expression of Ras guanyl nucleotide-releasing protein 1 in a subset of patients with systemic lupus erythematosus. J Immunol (2007) 179(7):4890–900. 10.4049/jimmunol.179.7.4890 17878389

[39] Dai MS Lu H. Crosstalk between c-Myc and ribosome in ribosomal biogenesis and cancer. J Cell Biochem (2008) 105(3):670–7. 10.1002/jcb.21895 18773413 PMC2569974

[40] Fanidi A Harrington EA Evan GI. Cooperative interaction between c-myc and bcl-2 proto-oncogenes. Nature (1992) 359(6395):554–6. 10.1038/359554a0 1406976

[41] Wagner AJ Small MB Hay N. Myc-mediated apoptosis is blocked by ectopic expression of Bcl-2. Mol Cell Biol (1993) 13(4):2432–40. 10.1128/MCB.13.4.2432 8455620 PMC359564

[42] Evan GI Vousden KH Proliferation, cell cycle and apoptosis in cancer. Nature (2001) 411(6835):342–8. 10.1038/35077213 11357141

[43] Yu L Fang F Dai X Xu H Qi X Fang M MKL1 defines the H3K4Me3 landscape for NF-kappaB dependent inflammatory response. Sci Rep (2017) 7(1):191 10.1038/s41598-017-00301-w 28298643 PMC5428227

[44] Shi L Song L Marurer K Sharp JG Zhang Z Sullivan K Endotoxin tolerance in monocytes can be mitigated by a2-interferon. J Leukoc Biol (2015) 98:651–9. 10.1189/jlb.4A0914-450RR 26206900 PMC4763867

[45] Garrett-Sinha LA Kearly A Satterthwaite AB. The role of the transcription factor Ets1 in lupus and other autoimmune diseases. Crit Rev Immunol (2016) 36(6):485–510. 10.1615/CritRevImmunol.2017020284 28845756 PMC5695541

[46] Sullivan KE Piliero LM Dharia T Goldman D Petri MA. 3′ polymorphisms of ETS1 are associated with different clinical phenotypes in SLE. Hum Mutat (2000) 16(1):49–53. 10.1002/1098-1004(200007)16:1<49:AID-HUMU9>3.0.CO;2-Z 10874305

[47] Li J Zhao S Yi M Hu X Li J Xie H Activation of JAK-STAT1 signal transduction pathway in lesional skin and monocytes from patients with systemic lupus erythematosus. Zhong Nan Da Xue Xue Bao Yi Xue Ban (2011) 36(2):109–15. 10.3969/j.issn.1672-7347.2011.02.003 21368418

[48] Smith CK Seto NL Vivekanandan-Giri A Yuan W Playford MP Manna Z Lupus high-density lipoprotein induces proinflammatory responses in macrophages by binding lectin-like oxidised low-density lipoprotein receptor 1 and failing to promote activating transcription factor 3 activity. Ann Rheum Dis (2017) 76(3):602–11. 10.1136/annrheumdis-2016-209683 27543414 PMC6109980

[49] Labzin LI Schmidt SV Masters SL Beyer M Krebs W Klee K ATF3 is a key regulator of macrophage IFN responses. J Immunol (2015) 195(9):4446–55. 10.4049/jimmunol.1500204 26416280

[50] Boehlk S Fessele S Mojaat A Miyamoto NG Werner T Nelson EL ATF and Jun transcription factors, acting through an Ets/CRE promoter module, mediate lipopolysaccharide inducibility of the chemokine RANTES in monocytic Mono Mac 6 cells. Eur J Immunol (2000) 30(4):1102–12. 10.1002/(SICI)1521-4141(200004)30:4<1102::AID-IMMU1102>3.0.CO;2-X 10760799

[51] Li QZ Zhou J Lian Y Zhang B Branch VK Carr-Johnson F Interferon signature gene expression is correlated with autoantibody profiles in patients with incomplete lupus syndromes. Clin Exp Immunol (2010) 159(3):281–91. 10.1111/j.1365-2249.2009.04057.x 19968664 PMC2819494

[52] Hung T Pratt GA Sundararaman B Townsend MJ Chaivorapol C Bhangale T The Ro60 autoantigen binds endogenous retroelements and regulates inflammatory gene expression. Science (2015) 350(6259):455–9. 10.1126/science.aac7442 26382853 PMC4691329

[53] Liu L Yin X Wen L Yang C Sheng Y Lin Y Several critical cell types, tissues, and pathways are implicated in genome-wide association studies for systemic lupus erythematosus. G3 (Bethesda) (2016) 6(6):1503–11. 10.1534/g3.116.027326 27172182 PMC4889647

[54] Farh KK Marson A Zhu J Kleinewietfeld M Housley WJ Beik S Genetic and epigenetic fine mapping of causal autoimmune disease variants. Nature (2015) 518(7539):337–43. 10.1038/nature13835 25363779 PMC4336207

[55] Wang D John SA Clements JL Percy DH Barton KP Garrett-Sinha LA. Ets-1 deficiency leads to altered B cell differentiation, hyperresponsiveness to TLR9 and autoimmune disease. Int Immunol (2005) 17(9):1179–91. 10.1093/intimm/dxh295 16051621

[56] Pan HF Leng RX Tao JH Li XP Ye DQ. Ets-1: a new player in the pathogenesis of systemic lupus erythematosus? Lupus (2011) 20(3):227–30. 10.1177/0961203310389842 21362749

