# Supplementary material for: Overall Downregulation of mRNAs and Enrichment of H3K4me3 Change Near Genome-Wide Association Study Signals in Systemic Lupus Erythematosus: Cell-Specific Effects
Source: Front Immunol. 2018 Mar 13;9:497. doi: 10.3389/fimmu.2018.00497 (PMC5859352; doi:10.3389/fimmu.2018.00497)
Supplement: Supplementary file 1 [file Data_Sheet_1.DOCX]

***Supplemental material***

**Overall Downregulation of mRNAs And Enrichment of H3K4me3 Change Near GWAS Signals In SLE: Cell Specific Effects**

**Zhe Zhang^a^, Lihua Shi^b^, Li Song^b^, Kelly Maurer^b^, Michele A Petri^c^, Kathleen E. Sullivan^b*^**

1. Supplementary Figures and Tables

1.1 Supplemental Tables

**Supplemental Table 1:** Expression gene sets

| **ID** | **Source** | **Name** | **Size** | **B_Change** | **B_Score** | **B_Pvalue** | **M_Change** | **M_Score** | **M_Pvalue** | **T_Change** | **T_Score** | **T_Pvalue** |
| --- | --- | --- | --- | --- | --- | --- | --- | --- | --- | --- | --- | --- |
| hsa04612 | KEGG | Antigen processing and presentation | 77 | **-62.4%** | **-3.8398** | **1.23E-04** | -36.7% | -2.5843 | 9.76E-03 | -37.6% | -2.6205 | 8.78E-03 |
| GO:0060326 | GO_BP | cell chemotaxis | 177 | **-68.4%** | **-7.888** | **3.11E-15** | +16.2% | 3.165 | 1.55E-03 | -5.2% | -0.48447 | 6.28E-01 |
| GO:0006935 | GO_BP | chemotaxis | 491 | **-37.6%** | **-9.0409** | **0.00E+00** | +5.3% | 1.0651 | 2.87E-01 | -6.1% | -2.1051 | 3.53E-02 |
| GO:0019955 | GO_MF | cytokine binding | 104 | **-35.7%** | **-3.9784** | **6.94E-05** | **+25.9%** | **4.003** | **6.25E-05** | +10% | 1.7659 | 7.74E-02 |
| h_all_19 | MSigDB | HALLMARK_INTERFERON_GAMMA_RESPONSE | 200 | **-33%** | **-5.2561** | **1.47E-07** | -18.8% | -3.2729 | 1.06E-03 | +10.3% | 3.7345 | 1.88E-04 |
| h_all_29 | MSigDB | HALLMARK_MYC_TARGETS_V2 | 58 | **+49.4%** | **5.5828** | **2.37E-08** | -12.2% | -0.74949 | 4.54E-01 | **+39.8%** | **4.3668** | **1.26E-05** |
| h_all_1 | MSigDB | HALLMARK_TNFA_SIGNALING_VIA_NFKB | 200 | **-103.4%** | **-12.669** | **0.00E+00** | -8.3% | -2.1653 | 3.04E-02 | **-59.5%** | **-8.2087** | **2.22E-16** |
| GO:0002764 | GO_BP | immune response-regulating signaling pathway | 493 | **-37.5%** | **-8.3566** | **0.00E+00** | **-13.9%** | **-4.2852** | **1.83E-05** | -7% | -0.34143 | 7.33E-01 |
| C0021053_All | DisGeNET | Immune System Diseases | 194 | **-62.6%** | **-7.559** | **4.06E-14** | **-21.4%** | **-4.2292** | **2.35E-05** | -15.8% | -2.8299 | 4.66E-03 |
| R-HSA-5663205 | REACTOME | Infectious disease | 448 | **+15.9%** | **11.169** | **0.00E+00** | +1.8% | 4.1594 | 3.19E-05 | **+48.7%** | **18.169** | **0.00E+00** |
| C1290884_All | DisGeNET | Inflammatory disorder | 235 | **-53.9%** | **-5.9716** | **2.35E-09** | -2.9% | -1.3337 | 1.82E-01 | -12.8% | -2.614 | 8.95E-03 |
| GO:0004908 | GO_MF | interleukin-1 receptor activity | 7 | -112.2% | -1.9135 | 5.57E-02 | +143.9% | 3.111 | 1.86E-03 | +60.2% | 0.69633 | 4.86E-01 |
| GO:0071674 | GO_BP | mononuclear cell migration | 44 | -90.7% | -2.9153 | 3.55E-03 | +55.8% | 2.3406 | 1.93E-02 | +14.2% | 0.42876 | 6.68E-01 |
| GO:0006397 | GO_BP | mRNA processing | 448 | **+27.5%** | **15.366** | **0.00E+00** | **+7.8%** | **6.2547** | **3.98E-10** | **+16.9%** | **5.554** | **2.79E-08** |
| GO:0034470 | GO_BP | ncRNA processing | 408 | **+59%** | **22.404** | **0.00E+00** | **+10.6%** | **8.1821** | **2.22E-16** | **+64.2%** | **19.691** | **0.00E+00** |
| GO:0000790 | GO_CC | nuclear chromatin | 335 | -13.4% | -1.2606 | 2.07E-01 | **-17.5%** | **-7.4346** | **1.05E-13** | **-19.3%** | **-6.8204** | **9.08E-12** |
| hsa05340 | KEGG | Primary immunodeficiency | 37 | -27.4% | -1.6958 | 8.99E-02 | **-51.9%** | **-4.5453** | **5.48E-06** | +7.8% | -0.019425 | 9.85E-01 |
| GO:0035455 | GO_BP | response to interferon-alpha | 20 | -30.3% | -1.0834 | 2.79E-01 | -12% | 0.37133 | 7.10E-01 | +60.9% | 3.0876 | 2.02E-03 |
| hsa03013 | KEGG | RNA transport | 171 | **+36.1%** | **10.002** | **0.00E+00** | +6.1% | 3.4832 | 4.96E-04 | **+28.8%** | **7.2121** | **5.51E-13** |

Red font indicates significant p values.

**Supplemental Table 2:** Common gene set enrichment categories between expression and H3K4me3

| **ID** | **Collection** | **Name** | **Size** | **B_Change_RNA** | **B_Score_RNA** | **B_Pvalue_RNA** | **B_Change_K4me3** | **B_Score_K4me3** | **B_Pvalue_K4me3** | **M_Change_RNA** | **M_Score_RNA** | **M_Pvalue_RNA** | **M_Change_K4me3** | **M_Score_K4me3** | **M_Pvalue_K4me3** | **T_Change_RNA** | **T_Score_RNA** | **T_Pvalue_RNA** | **T_Change_K4me3** | **T_Score_K4me3** | **T_Pvalue_K4me3** |
| --- | --- | --- | --- | --- | --- | --- | --- | --- | --- | --- | --- | --- | --- | --- | --- | --- | --- | --- | --- | --- | --- |
| **BIOCARTA_110** | **C2_BioCarta_Pathways** | **BIOCARTA_TCRA_PATHWAY** | **13** | -183.06% | -3.7776 | 1.60E-04 | -45.4% | -3.2559 | 1.10E-03 | **-144.55%** | **-4.9795** | **6.40E-07** | **-81.5%** | **-6.4578** | **1.10E-10** | -8.67% | -0.0957 | 9.20E-01 | -18.1% | -0.9853 | 3.20E-01 |
| **GO:0022403** | **GO_BP** | **cell cycle phase** | **342** | **+8.38%** | **5.3942** | **6.90E-08** | +1.89% | 2.5014 | 1.20E-02 | -8.64% | -1.8042 | 7.10E-02 | **+5.19%** | **6.3605** | **2.00E-10** | **+20.58%** | **8.1209** | **4.40E-16** | **+4.9%** | **6.4839** | **8.90E-11** |
| **GO:0051301** | **GO_BP** | **cell division** | **479** | -2.26% | 0.5136 | 6.10E-01 | +2.74% | 3.7721 | 1.60E-04 | -7.53% | -3.2852 | 1.00E-03 | +2.46% | 3.9776 | 7.00E-05 | **+9.43%** | **4.209** | **2.60E-05** | **+2.46%** | **4.4183** | **9.90E-06** |
| **GO:0032990** | **GO_BP** | **cell part morphogenesis** | **461** | **-14.99%** | **-5.7458** | **9.20E-09** | **+7.18%** | **6.9673** | **3.20E-12** | -6.25% | -3.038 | 2.40E-03 | -2.53% | -3.0324 | 2.40E-03 | -10.19% | -3.5444 | 3.90E-04 | +2.03% | 2.1636 | 3.00E-02 |
| **GO:0006281** | **GO_BP** | **DNA repair** | **500** | **+11.31%** | **8.1942** | **2.20E-16** | +2.31% | 2.7987 | 5.10E-03 | -6.19% | -2.1243 | 3.40E-02 | **+3.1%** | **4.7443** | **2.10E-06** | **+11.73%** | **4.6549** | **3.20E-06** | **+4.68%** | **6.358** | **2.00E-10** |
| **GO:0007163** | **GO_BP** | **establishment or maintenance of cell polarity** | **155** | **-23.34%** | **-5.8007** | **6.60E-09** | **+5.7%** | **4.072** | **4.70E-05** | **-14.3%** | **-6.0508** | **1.40E-09** | -1.26% | -0.2187 | 8.30E-01 | -11.73% | -2.9773 | 2.90E-03 | +1.82% | 1.4701 | 1.40E-01 |
| **h_all_1** | **C0_Hallmark** | **HALLMARK_TNFA_SIGNALING_VIA_NFKB** | **200** | **-103.37%** | **-12.669** | **0.00E+00** | -4.03% | -1.3279 | 1.80E-01 | -8.31% | -2.1653 | 3.00E-02 | **+12.51%** | **9.518** | **0.00E+00** | **-59.11%** | **-8.2087** | **2.20E-16** | **+6%** | **6.3643** | **2.00E-10** |
| **R-HSA-3214815** | **REACTOME** | **HDACs deacetylate histones** | **94** | -37.44% | -3.3957 | 6.80E-04 | +10.19% | 3.5211 | 4.30E-04 | -17.98% | -2.603 | 9.20E-03 | +7.92% | 3.5325 | 4.10E-04 | **-39.47%** | **-3.9345** | **8.30E-05** | **+8.67%** | **3.9842** | **6.80E-05** |
| **GO:0006954** | **GO_BP** | **inflammatory response** | **490** | **-57%** | **-10.073** | **0.00E+00** | **-6.81%** | **-4.9026** | **9.50E-07** | +0.95% | -0.4332 | 6.60E-01 | **+4.83%** | **5.3937** | **6.90E-08** | -13.29% | -3.2336 | 1.20E-03 | -4.1% | -3.5088 | 4.50E-04 |
| **R-HSA-6783783** | **REACTOME** | **Interleukin-10 signaling** | **49** | **-168.16%** | **-5.2517** | **1.50E-07** | **-19.75%** | **-4.6998** | **2.60E-06** | +33.18% | 3.6048 | 3.10E-04 | **+18.92%** | **6.0741** | **1.20E-09** | -26.58% | -1.3871 | 1.70E-01 | -8.67% | -2.4061 | 1.60E-02 |
| **GO:0045321** | **GO_BP** | **leukocyte activation** | **471** | **-39.87%** | **-8.567** | **0.00E+00** | **-4.83%** | **-3.9254** | **8.70E-05** | **-12.37%** | **-5.1513** | **2.60E-07** | **-5.78%** | **-4.3204** | **1.60E-05** | -7.92% | -1.7191 | 8.60E-02 | -4.17% | -3.0994 | 1.90E-03 |
| **GO:0050900** | **GO_BP** | **leukocyte migration** | **280** | **-53.84%** | **-9.2211** | **0.00E+00** | **-7.92%** | **-4.3838** | **1.20E-05** | +8.52% | 2.5546 | 1.10E-02 | +2.6% | 2.1025 | 3.60E-02 | -4.1% | -0.8698 | 3.80E-01 | -4.39% | -3.1832 | 1.50E-03 |
| **hsa04010** | **pathway** | **MAPK signaling pathway** | **255** | **-38.11%** | **-6.2488** | **4.10E-10** | **+5.48%** | **3.9724** | **7.10E-05** | **-14.53%** | **-3.9582** | **7.60E-05** | -0.5% | -0.3667 | 7.10E-01 | **-23.97%** | **-6.0235** | **1.70E-09** | +2.38% | 1.9259 | 5.40E-02 |
| **GO:0006397** | **GO_BP** | **mRNA processing** | **448** | **+27.46%** | **15.366** | **0.00E+00** | +0.36% | 0.2403 | 8.10E-01 | **+7.85%** | **6.2547** | **4.00E-10** | +2.6% | 3.685 | 2.30E-04 | **+16.47%** | **5.554** | **2.80E-08** | **+3.53%** | **5.233** | **1.70E-07** |
| **GO:0000790** | **GO_CC** | **nuclear chromatin** | **335** | -13.39% | -1.2606 | 2.10E-01 | +3.6% | 3.6074 | 3.10E-04 | **-17.48%** | **-7.4346** | **1.00E-13** | +1.82% | 2.1548 | 3.10E-02 | **-18.92%** | **-6.8204** | **9.10E-12** | **+4.61%** | **5.0556** | **4.30E-07** |
| **GO:0050715** | **GO_BP** | **positive regulation of cytokine secretion** | **97** | **-106.61%** | **-7.3474** | **2.00E-13** | **-12.51%** | **-4.7841** | **1.70E-06** | -6.16% | -1.6898 | 9.10E-02 | +3.24% | 1.4416 | 1.50E-01 | -24.83% | -1.0138 | 3.10E-01 | -5.92% | -2.7146 | 6.60E-03 |
| **hsa05340** | **pathway** | **Primary immunodeficiency** | **37** | -27.39% | -1.6958 | 9.00E-02 | -5.04% | -0.9603 | 3.40E-01 | **-51.93%** | **-4.5453** | **5.50E-06** | **-24.83%** | **-4.7231** | **2.30E-06** | +7.92% | -0.0194 | 9.80E-01 | -7.92% | -1.6693 | 9.50E-02 |
| **R-HSA-195258** | **REACTOME** | **RHO GTPase Effectors** | **295** | **-14.96%** | **-4.1037** | **4.10E-05** | +4.17% | 3.6401 | 2.70E-04 | **-13.78%** | **-4.5264** | **6.00E-06** | **+4.75%** | **4.6585** | **3.20E-06** | -5.26% | -0.3524 | 7.20E-01 | **+4.97%** | **5.4562** | **4.90E-08** |
| **hsa03013** | **pathway** | **RNA transport** | **171** | **+36.07%** | **10.002** | **0.00E+00** | +3.96% | 2.0661 | 3.90E-02 | +6.14% | 3.4832 | 5.00E-04 | +2.81% | 1.7759 | 7.60E-02 | **+28.34%** | **7.2121** | **5.50E-13** | +4.9% | 3.3968 | 6.80E-04 |
| **hsa04660** | **pathway** | **T cell receptor signaling pathway** | **105** | -38.79% | -3.8404 | 1.20E-04 | -4.03% | -0.7984 | 4.20E-01 | **-22.57%** | **-4.1411** | **3.50E-05** | **-13.29%** | **-4.5275** | **6.00E-06** | -7.92% | -1.6803 | 9.30E-02 | +0.3% | 1.1774 | 2.40E-01 |
| **lymph_node** | **TiGER** | **Tissue-specific expression in lymph_node** | **413** | +1.23% | -1.6389 | 1.00E-01 | +0.63% | -0.6552 | 5.10E-01 | **-16.24%** | **-6.6959** | **2.10E-11** | **-6.29%** | **-4.0677** | **4.70E-05** | -6.22% | -2.2255 | 2.60E-02 | **-7.92%** | **-5.0072** | **5.50E-07** |

Red font indicates significant p values for increases and green font indicates significant p values for decreases.

1.2 Supplementary figure


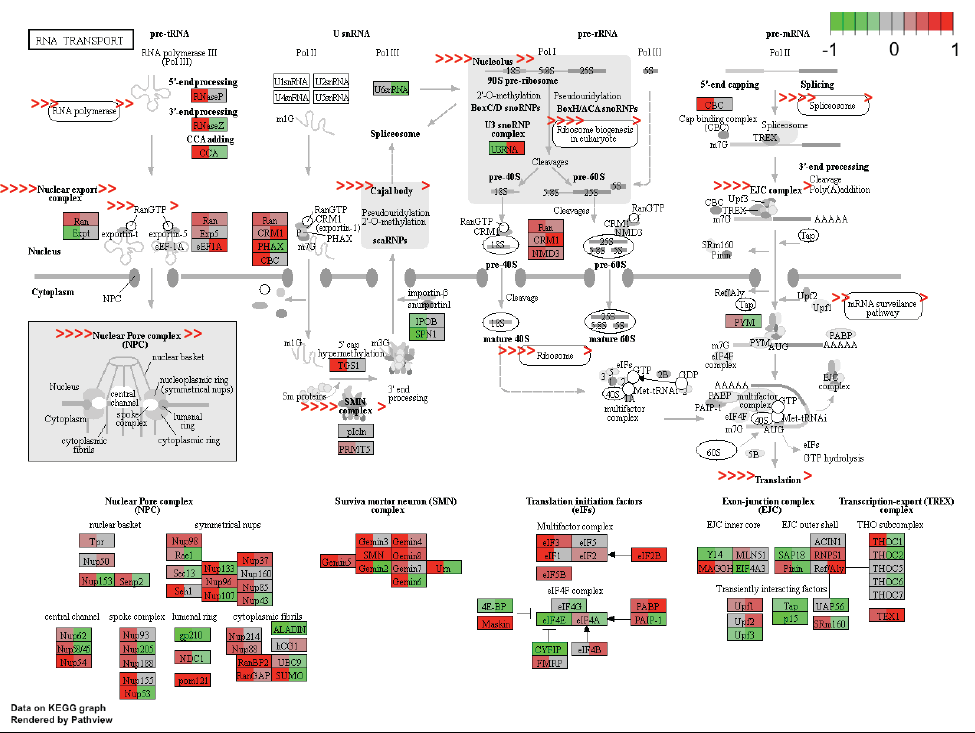


**Supplementary Figure 1.** RNA transport genes had both increased expression and increased H3K4me3. They can be viewed as part of a landscape of altered gene expression in SLE. Red indicates increased expression.
